# A gratuitous β-Lactamase inducer uncovers hidden active site dynamics of the *Staphylococcus aureus* BlaR1 sensor domain

**DOI:** 10.1371/journal.pone.0197241

**Published:** 2018-05-17

**Authors:** Thomas E. Frederick, Jeffrey W. Peng

**Affiliations:** 1 Department of Chemistry and Biochemistry, University of Notre Dame, Notre Dame, IN, United States of America; 2 Department of Physics, University of Notre Dame, Notre Dame, IN, United States of America; University of Pittsburgh School of Medicine, UNITED STATES

## Abstract

Increasing evidence shows that active sites of proteins have non-trivial conformational dynamics. These dynamics include active site residues sampling different local conformations that allow for multiple, and possibly novel, inhibitor binding poses. Yet, active site dynamics garner only marginal attention in most inhibitor design efforts and exert little influence on synthesis strategies. This is partly because synthesis requires a level of atomic structural detail that is frequently missing in current characterizations of conformational dynamics. In particular, while the identity of the mobile protein residues may be clear, the specific conformations they sample remain obscure. Here, we show how an appropriate choice of ligand can significantly sharpen our abilities to describe the interconverting binding poses (conformations) of protein active sites. Specifically, we show how 2-(2’-carboxyphenyl)-benzoyl-6-aminopenicillanic acid (CBAP) exposes otherwise hidden dynamics of a protein active site that binds β-lactam antibiotics. When CBAP acylates (binds) the active site serine of the β-lactam sensor domain of BlaR1 (BlaR^S^), it shifts the time scale of the active site dynamics to the slow exchange regime. Slow exchange enables direct characterization of inter-converting protein and bound ligand conformations using NMR methods. These methods include chemical shift analysis, 2-d exchange spectroscopy, off-resonance ROESY of the bound ligand, and reduced spectral density mapping. The active site architecture of BlaR^S^ is shared by many β-lactamases of therapeutic interest, suggesting CBAP could expose functional motions in other β-lactam binding proteins. More broadly, CBAP highlights the utility of identifying chemical probes common to structurally homologous proteins to better expose functional motions of active sites.

## Introduction

The discovery of penicillin and other β-lactam antibiotics is one of the most significant medical advances of the 20^th^ century [1]. However, a post-antibiotic world where simple bacterial infections kill unabated is increasingly likely. Exorbitant use of antibiotics, particularly β-lactams, has amplified resistance phenotypes among both gram-positive and gram-negative bacteria [2–4]. The severity of this resistance is highlighted by the rise of clinical isolates resistant to carbapenems, a class of β-lactams considered to be drugs of last resort [5].

β-lactams irreversibly bind and inhibit penicillin-binding proteins (PBPs), bacterial proteins that maintain the essential cell wall. Bacteria resist β-lactam action through multiple mechanisms, two of which include: (**i**) production of β-lactamase proteins that hydrolytically destroy the β-lactam drugs; and (**ii**) alteration (mutation) of the PBPs that reduce their affinity for β-lactams [6]. For gram-positive bacteria such as *Staphylococcus aureus* (*S*. *aureus*), the resistance phenotype comes primarily from plasmid born resistance factors, specifically Ambler class A β-lactamases and PBPs (e.g. TEM-1 β-lactamase and PBP2a respectively) [7–9]. These plasmids also contain gene clusters coding for β-lactam sensor/signal transducer proteins (BlaR1/MecR1) and transcriptional regulator factors (BlaI/MecI) [10]. These proteins regulate the expression of β-lactamases and PBPs, suppressing/activating their expression in the absence/presence of β-lactams. Interestingly, BlaR1 can act as the sensor and signal transducer for both the β-lactamase and PBP gene clusters, including the resistance protein PBP2a [11].

BlaR1 relays a signal, originating with a β-lactam binding its extracellular sensor domain (BlaR^S^), to its intracellular zinc protease domain [12]. The activated zinc protease degrades BlaI/MecI allowing for β-lactamase and PBP expression [13]. Because pathogenic gram-positive bacteria (e.g. methicillin-resistant *S*. *aureus* or MRSA) rely on the *bla* gene cluster, BlaR1 has become an important therapeutic target.

**Fig 1:**
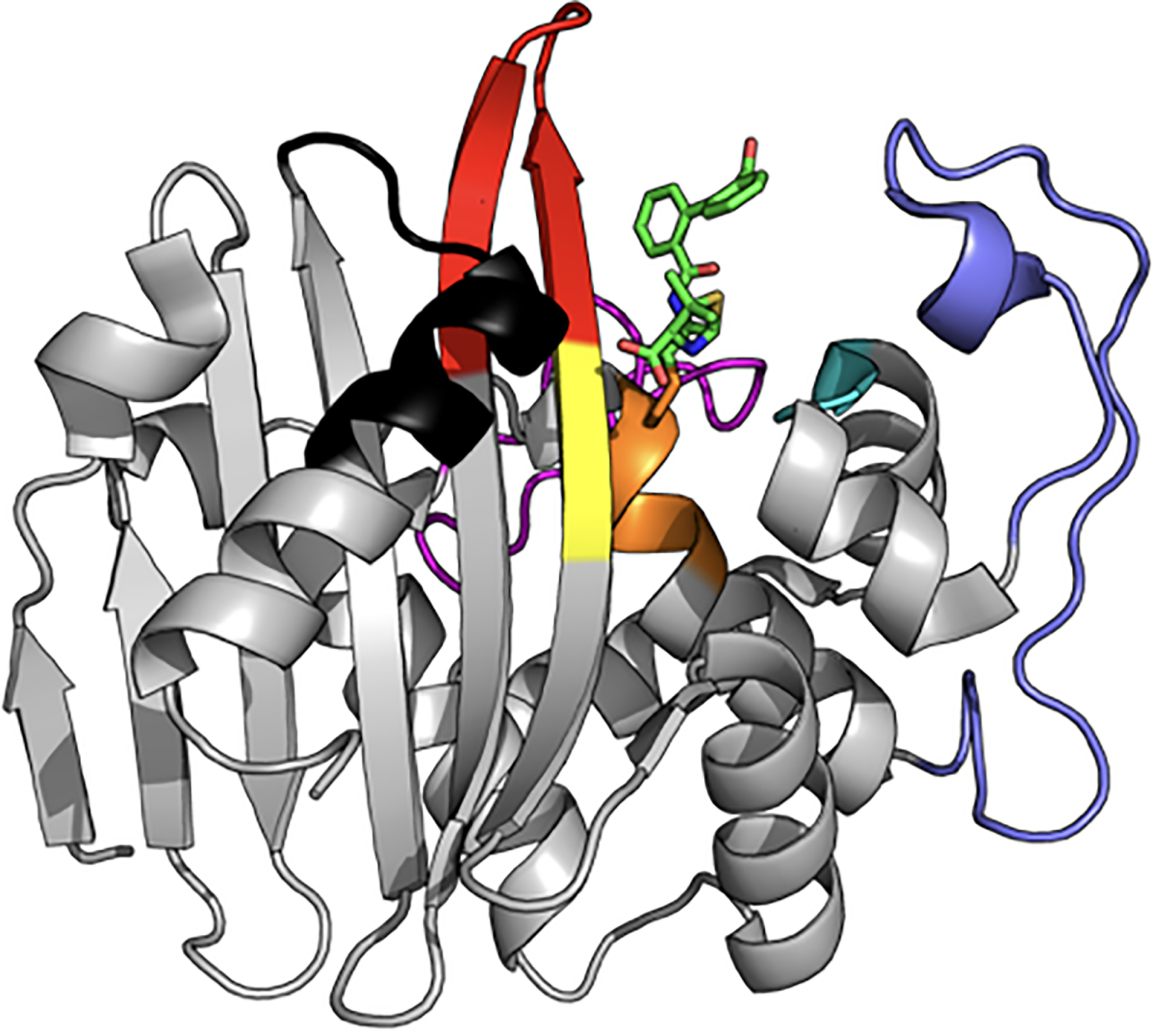
Structure of the CBAP-acylated BlaR1 sensor domain. Ribbon representation of BlaR^S^ acylated by CBAP (PDB code 3Q7Z). Three conserved structural features include the β5/β6 hairpin (red), the P-loop (slate), the Ω-loop (magenta). Conserved sequence motifs include the “S-x-x-K” (Orange, S389-T390-Y391-K392), the “S-x-N/D” (Cyan, S437-V438-N439), and the “K-T/S-G” (yellow, K526-T527-G528) motifs. The bound β-lactam CBAP is indicated by Green sticks. The β7-Helix K turn is indicated in black.

BlaR1 is a transmembrane protein with an extracellular C-terminal sensor domain (BlaR^S^, residues 330–585, Mw = 29,000) that resides on the cell surface [11,14,15]. The BlaR^S^ sensor domain is structurally homologous to the class D β-lactamases, with C^α^ RMSDs between 1.17 and 1.41 Å [16]. Both proteins are members of a larger super-family of acyl-transferases that share three conserved amino acid segments critical for β-lactam binding: (1) the “S-x-x-K”, (2) the “K-T/S-G”, and (3) the “S-x-N/D” motifs (Fig 1, orange, yellow, and cyan, respectively) [4,17]. These correspond to S389-T390-Y391-K392, K526-T527-G528, and S437-V438-N439 in *S*. *aureus* BlaR^S^ [17]. In the “S-x-x-K” motif, S389 is the site of acylation and K392 primes S389 for nucleophilic attack on the β-lactam ring. Just outside the antibiotic binding pocket, both BlaR^S^ and the Class D β-lactamases have three surface loops in common (albeit with greater sequence variability): the P-loop, the Ω-loop, and the β5-β6 hairpin (Fig 1, slate, magenta, and red respectively). Additionally, residues of Helix K (Fig 1, black) may participate in signal transduction [16].

BlaR^S^ senses β-lactams by forming a covalent acyl-enzyme complex with a longevity exceeding the bacterial doubling time. What remains unclear is how acylation perturbs the rest of BlaR1 for trans-membrane signal transduction. Two hypotheses, although not mutually exclusive, prevail: (1) Intramolecular contact between BlaR^S^ and extracellular loop 2 (L2) gates β-lactam access to the BlaR^S^ binding pocket [15,18,19] and (2) BlaR^S^ acylation induces a conformational change by altering secondary structure. Evidence supporting the first hypothesis consists of phage-display studies (*B*. *licheniformis*) [18,20], and our recent paramagnetic relaxation enhancement (PRE) studies (*S*. *aureus*) [18,20]. Evidence supporting the second hypothesis has been more tenuous.

The consensus among X-ray crystal structures is that BlaR^S^ acylation, regardless of the specific type of β-lactam, does not elicit a stark conformational change [16,19]. Solution spectroscopy has given a more plastic view: far-UV circular dichroism (CD) investigations of the *S*. *aureus* and *B*. *licheniformis* sensor domains indicate acylation induces enhanced secondary structure in *S*. *aureus* BlaR^S^, but not in *B*. *licheniformis* [15,18]. Furthermore, we recently reported NMR studies showing that acylation by penicillin G (penG) perturbs the backbone dynamics of BlaR^S^ on both the subnanosecond and microsecond-millisecond time scales [21]. These results notwithstanding, direct observation of acylation-induced conformational change has been lacking.

**Fig 2:**
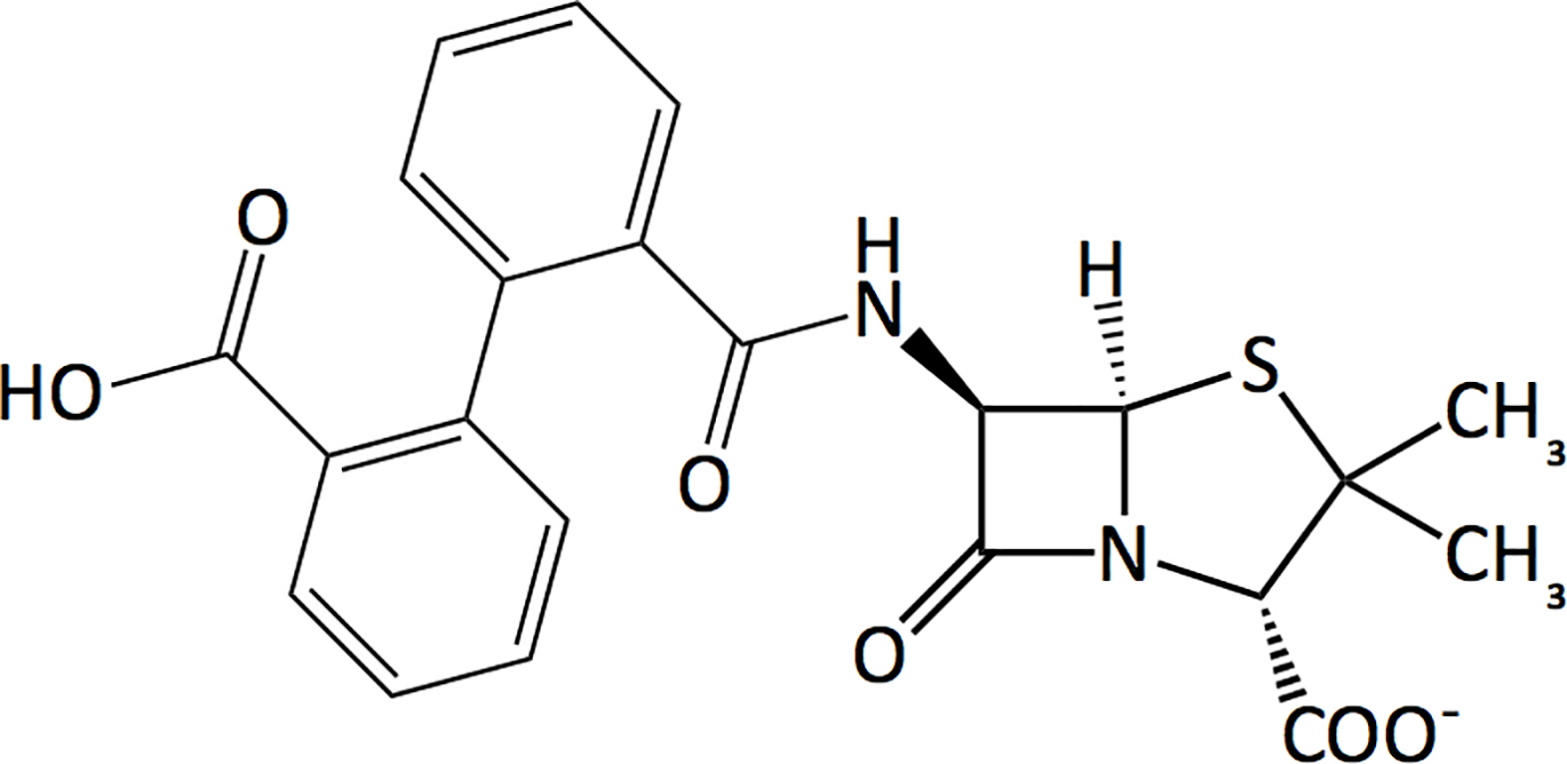
Chemical structure of CBAP.

Here, we report direct evidence of conformational change in BlaR^S^ upon acylation by the β-lactam 2-(2’-carboxyphenyl)-benzoyl-6-aminopenicillanic acid (CBAP, Fig 2). CBAP has been used to define the constituent events in BlaR1 signal transduction, including the auto-processing of the intracellular zinc protease domain and the degradation of BlaI that leads to β-lactamase expression [13,22]. CBAP has poor antimicrobial properties; nevertheless, it is among the best inducers of β-lactamase expression under control of the *bla* system [23–25]. Given CBAP’s exceptional induction of β-lactamase expression, we wanted to understand the atomic-level consequences of its acylation of BlaR^S^. Serendipitously, CBAP has given us the first direct NMR spectroscopic evidence that the BlaR^S^ antibiotic binding pocket undergoes slow exchange between distinct conformational states.

The results presented here strengthen the view that predicting the consequences of protein binding interactions requires consideration of the intrinsic conformational dynamics. This view has gained traction in large part due to robust NMR experiments that can identify protein regions experiencing conformational exchange. Such exchange involves amino acid residues sampling different local conformations with different intrinsic chemical shifts for a given amino acid spin system (e.g. a particular amide ^1^H-^15^N). Effectively, the exchange dynamics render the spin-system chemical shifts time-dependent; the results include changes in line-width and/or lineshape that depend on the exchange rapidity relative to the span (in hertz) of chemical shifts sampled by the exchange. Under slow exchange conditions (exchange rate constants smaller than the chemical shift span), direct observation of resonances from the exchanging states is possible. Typically, faster exchange conditions prevail such that an exchanging spin system produces a single resonance. In the latter scenario, information specific to the exchanging conformations, such as populations and chemical shifts, arrives indirectly via methods such relaxation dispersion, or paramagnetic relaxation enhancements. Such was the case for our previous study of BlaR^S^ acylated by Penicillin-G (PenG) [21].

Yet, direct observation of the resonances from the exchanging states would clearly be useful. For example, the ability to observe resonances of rare (high-energy) conformations could test for conformational-selection mechanisms mediating substrate recognition and catalysis. This was highlighted in recent work by Wolf-Watz and co-workers, in which strategic introduction of disulfide bridges to adenylate kinase trapped a rare apo state conformer that coincided with the catalytically relevant closed (substrate bound) conformation [26].

Here, our studies of BlaR^S^ demonstrate an alternative approach that exploits small molecule interactions. We show how small-molecule binding to a protein can act as a chemical “time-scale shifter” that exposes otherwise obscured conformational dynamics in proteins. Specifically, CBAP binding at the BlaR^S^ active site reveals separate sets of NMR resonances, attesting to slow conformational exchange between distinct bound states. Critically, the slow exchange condition enables direct characterization of previously obscure conformational states. Our results unequivocally establish the dynamic nature of the BlaR^S^ active site and its sensitivity to β-lactam binding. The revelation of these hidden conformations lays the groundwork for investigating alternative binding poses that may accelerate the design of new inhibitors of BlaR^S^ and its structural homologs. More broadly, our results highlight the potential of small molecule ligands as tools to more directly illuminate the conformations sampled by dynamic active sites.

## Materials and methods

### Materials

CBAP (2-(2’-carboxyphenyl)-benzoyl-6-aminopenicillanic acid) was generously provided by the Mobashery group (University of Notre Dame). All isotopes for protein expression were purchased from Cambridge Isotope Laboratories, Inc.

### Protein expression and purification

We expressed BlaR^S^ using BL21 (DE3) E. *coli* cells (Novagen) transformed with pET28a(+) plasmid (Novagen) coding for the extracellular sensor domain of S. *aureus* BlaR1 (residues 329–585). Perdeuterated U[^15^N,^13^C]-BlaR^S^ for all NMR ^15^N relaxation experiments was expressed in M9 media according to Marley *et al* as described previously [21]. U[^2^D]-BlaR^S^ for bound CBAP experiments was expressed using CELTONE media. Specifically, the pellet of a 5 mL Luria broth overnight was suspended in 250 mL of M9 media (99% D_2_O) containing ^14^NH_4_ (1 g/L), U[^2^D, ^12^C] D-glucose (1.5 g/L), and U[^2^D,^12^C,^14^N] Celtone Base Powder (1 g/L) in addition to thiamine, MgSO_4_, and CaCl_2_. The cells were incubated at 37 °C and 240 rpm until OD_600_ ≈ 0.7, then an additional 30 minutes at 24.1 °C and 225 rpm. Cells were induced with IPTG and incubated overnight (18–21 hours) prior to harvesting at 4,000 rpm for 20 minutes.

Purification of isotopically enriched BlaR^S^ followed previously established procedures [21]. NMR samples were prepared in either H_2_O or D_2_O BlaR^S^ NMR buffer (20 mM sodium phosphate dibasic, 30 mM NaCl, 0.02% NaN_3_, 10% D_2_O pH 7.0 or 99.9% D_2_O pD 7.0). Sample concentrations were generally 250 μM and loaded in magnetic susceptibility matched Shigemi tubes to reduce the necessary sample volume.

### Sequential NMR assignments

Backbone ^1^H^N^ and ^15^N resonances were sequentially assigned using standard TROSY-based triple-resonance spectra (3D HNCA/HNCOCA, HNCACO/HNCO, and HNCACB/HNCACB [27–29]) recorded at 16.4 T **(**700.13 MHz) and 295 K using a Bruker Avance system equipped with a cryogenically cooled TCI probe. NMR spectra were processed using TOPSPIN 1.3 (Bruker Biospin, Inc.), and sequential assignments were aided by SPARKY-3 [30] and CARA [31]. The magnitude of ^15^N-^1^H^N^ chemical shift perturbations (CSPs) were calculated as:

$$\Delta \delta_{total}=\sqrt{{\left(\Delta \delta_{H}\right)}^{2}+{\left(0.154\times \Delta \delta_{N}\right)}^{2}}$$ (1)

Here, Δδ_H_ and Δδ_N_ are the changes in the ^1^H^N^ and ^15^N chemical shifts; the 0.154 weighting factor corresponds to the average Δδ_H_/Δδ_N_ ratio among chemical shifts recorded in the BMRB database [32].

### NMR relaxation experiments

Amide ^15^N relaxation parameters R_1_ = 1/T_1_, R_2_ = 1/T_2_, and the steady-state heteronuclear ^1^H^N^-^15^N NOEs (ssNOE) were measured on an 18.8 T (800 MHz) Bruker Avance system installed with a cryogenically cooled TCI probe. The R_1_ relaxation delays were: 0.096 (twice), 0.304, 0.496, 0.704, 0.896, 1.104, 1.296 and 1.504 s. We used the water flip-back scheme of Chen and Tjandra to maintain water magnetization on the +z axis during the relaxation delay to minimize radiation dampening while suppressing cross correlation artifacts [33]. The R_2_ delays included 8.16 (twice), 16.32, 24.48, 32.64, 40.80, 48.96 and 57.12 ms using the CPMG pulse-scheme [34–36] with 900 μs between consecutive ^15^N refocusing pulses. The ^1^H^N^-^15^N ssNOE were determined using 5 s of ^1^H saturation per Ferrage *et al*. [37]. Relaxation rate constants were fitted using standard Levenburg-Marquardt procedures [38]. Statistical uncertainties were estimated using a jack-knife strategy and a repeat time-point to estimate the peak integration error within each experiment.

2D ^1^H^N^-^15^N exchange spectroscopy (EXSY) was performed using a modified ^15^N R_1_ experiment in which ^15^N chemical shift labeling preceded the longitudinal exchange (relaxation) delay [39,40]. The exchange delays included: 0.02, 0.10, 0.20, 0.30, 0.40, 0.50, 0.60, 0.70, 0.80, 1.00, 1.25, and 1.55 s. We fit the time-dependent ratios of the exchange and diagonal cross-peak intensities to the two-state EXSY expressions of Ernst *et al*. [41] to estimate the exchange rate constants. Statistical uncertainties in the rate constant were estimated via Monte Carlo simulations based on spectral duplicates.

To compare site specific flexibility along the BlaR^S^ backbone, we used J_eff_(0), the zero-frequency value of the NH spectral density function, determined from the ^15^N relaxation parameters via [42,43]:

$$J_{eff}\left(0\right)=\frac{3}{2\left(3D+C\right)}\left(R_{2,eff}-\frac{R_{1}}{2}-\frac{3\sigma_{NH}}{5}\right)$$ (2)

The constants $C=\Delta^{2}\omega_{N}^{2}/3$ and $D=\hbar^{2}\gamma_{H}^{2}\gamma_{N}^{2}/\langle r_{NH}^{6}\rangle$ in Eq 2 correspond to the ^15^N chemical shift anisotropy and ^1^H^N^-^15^N dipolar relaxation mechanisms, respectively. Here *γ*_*H*_ and *γ*_*N*_ are the proton and nitrogen gyromagnetic ratios. The heteronuclear dipolar cross-relaxation rate *σ*_*NH*_ was determined from the longitudinal relaxation rate R_1_ and ssNOE:

$$\sigma_{NH}=ssNOE\times R_{1}\times \frac{\gamma_{N}}{\gamma_{H}}$$ (3)

The “eff” subscript indicates effective J(0) values that include the possible contribution of exchange broadening sensed during R_2_ measurements. Specifically J_eff_(0) = J(0) + R_ex_, where J(0) is the inherent value reflecting nanosecond-subnanosecond re-orientational NH bond motions, and R_ex_ is the contribution of μs-ms chemical exchange processes. To minimize contributions from the overall protein tumbling when comparing apo versus CBAP-acylated BlaR^S^ J_eff_(0), we used the dimensionless ratio described by Eq 4 [21]:

$$\frac{\Delta J_{eff}\left(0\right)}{J_{eff}^{apo}\left(0\right)}=\frac{J_{eff}^{CBAP}\left(0\right)-J_{eff}^{apo}\left(0\right)}{J_{eff}^{apo}\left(0\right)}$$ (4)

NHs whose dimensionless ratio extend beyond one standard deviation of the trimmed mean, within error, were taken as having significant changes in backbone flexibility.

### NMR of bound CBAP

We used U-[^2^D,^12^C,^14^N] BlaR^S^ to record standard 2-D ^1^H-^1^H NOESY, TOCSY and ROESY spectra [44] of bound ligand. Spectra were recorded at 18.8 T and T(nom) = 293.8 K. We used an 80 ms NOESY mixing time and a 35 ms DIPSI-2 TOCSY spin-lock (ν = ω_1_/2π = 6,950 Hz). Off-resonance ^1^H-^1^H ROESY spectra of bound CBAP were obtained using a 20 ms spin lock (ν = ω_1_/2π = 7,000 Hz) applied off resonance at 20.72 ppm corresponding to a 35° tilt angle for spins on resonance at 8.36 ppm. For slowly tumbling molecules, this tilt angle nullifies the effective dipolar cross-relaxation rate constant; therefore, cross peaks reflect pure exchange [45,46]. The crystal structure PDB 3Q7Z of CBAP-acylated BlaR^S^ aided the assignments of bound CBAP resonances.

## Results

CBAP provides a glimpse into the conformational dynamics of BlaR^S^ beyond the reach of other β-lactam antibiotics. Specifically, acylation of BlaR^S^ by CBAP introduces slow conformational exchange in which the resonances of the interconverting states become directly observable. In the present study of CBAP, we have exploited this unique opportunity to directly study the exchange-coupled states and gain fresh insight into the conformational plasticity of the BlaR^S^ antibiotic binding pocket.

To ensure BlaR^S^ remained acylated throughout the NMR experiments we used an approximately ten-fold molar excess of CBAP (3 mM) over BlaR^S^ (0.25 mM). We observed NMR spectral features characteristic of active-site acylation, which includes peak splitting of residue G534 (S1A Fig) and reduced intensity of the side chain ^1^H^N^-^15^N^ζ^ resonance corresponding to the carboxylated active site lysine K392 (S1B Fig). The reduced intensity reflects K392 N^ζ^-decarboxylation critical for formation of a stable CBAP-acylated complex with a lifetime exceeding the bacterial doubling time [22,47,48]. Deacylation, indicated by the return of the G534 ^1^H^N^-^15^N resonance indicative of apo BlaR^S^, was detected only after ~ 1 month following the initial CBAP addition (S1A Fig). Combined, these findings indicate persistence of the covalent acyl-protein adduct as opposed to the non-covalent protein-substrate complex. Importantly, this means that the chemical exchange processes reported herein correspond to covalently bound ligand and/or protein dynamics rather than a [*P*]+[*L*] [*PL*] binding equilibrium.

### Chemical shift perturbations in BlaR^S^ upon CBAP acylation

**Fig 3:**
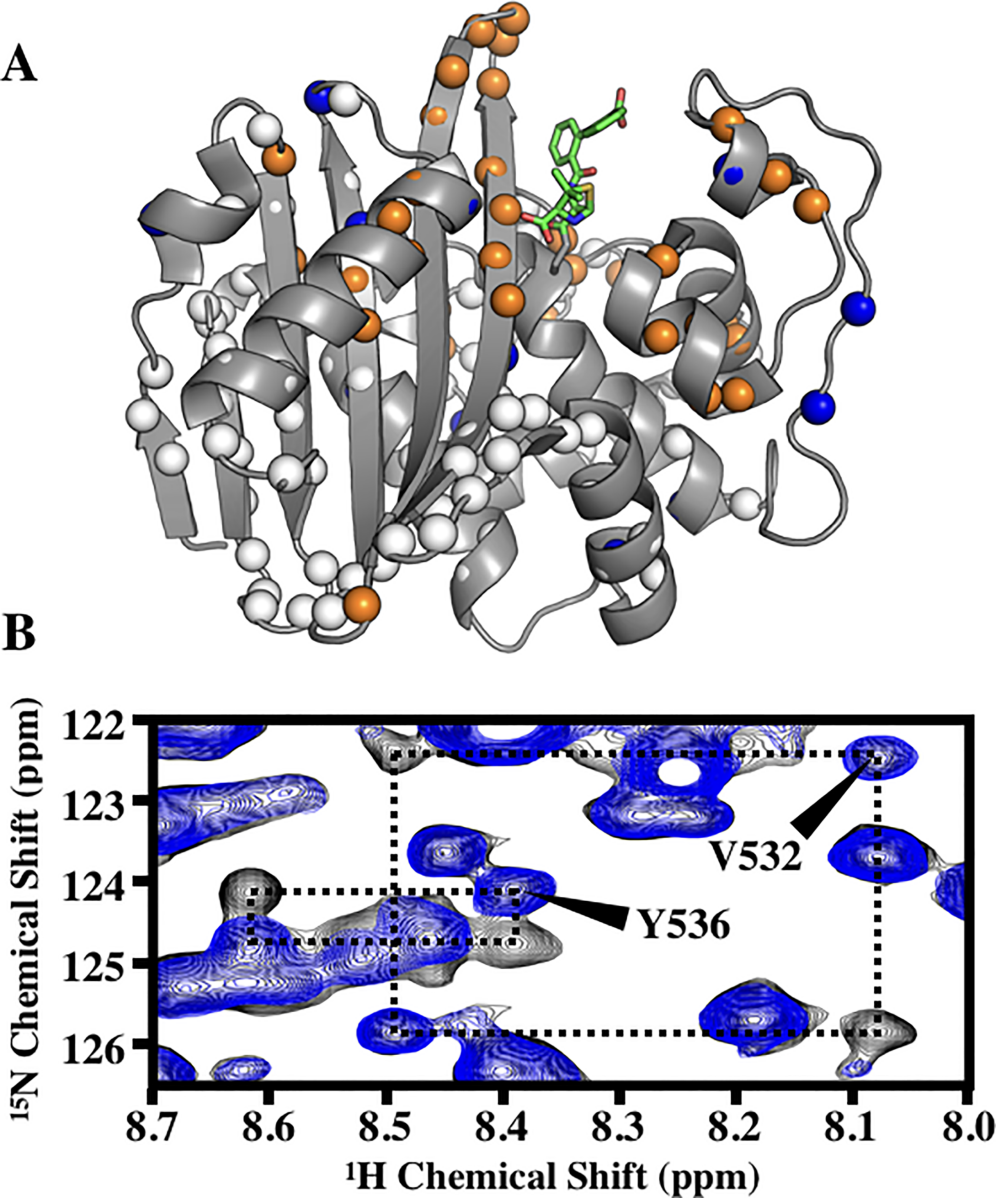
Slow exchange and amide CSPs from CBAP-acylation of BlaR^S^. (A) Residues demonstrating slow chemical exchange (orange spheres) and significant CSPs (blue spheres) are mapped onto PDB 3Q7Z. Spheres indicate residues assigned in both apo and CBAP-acylated BlaR^S^. Green sticks represent the bound β-lactam CBAP. (B) Pronounced ^15^N^H^-^1^H exchange squares of V532 and Y536 of U-[^15^N] 80% deuterated CBAP-acylated BlaR^S^. Spectra were recorded at 18.8 T and T(nom) = 293.8 K; ^15^N TROSY (Blue) versus EXSY-R_1_ TROSY (black) using a 400 ms exchange relaxation delay.

CBAP-acylated BlaR^S^ leads to prominent backbone amide chemical shift perturbations (CSPs) relative to the apo protein (Fig 3A, orange/blue spheres, corresponding bar plot in S2A Fig). CSPs beyond two standard deviations of the trimmed mean (0.022 ± 0.016 ppm) were considered significant. Most CSPs occurred within 15 Å of the bound CBAP molecule as seen in the crystal structure (PDB 3Q7Z), with prominent CSPs in the β5/β6-hairpin and the N-terminus of Helix K. These likely reflect ring currents of the CBAP phenyl substituents. More distal CSPs include conserved residues in hinge 1 of the Ω-loop (Y463, N465), residues in helices A and G (K354 and T454/A455 respectively), and N548 of the distal β6/β7-hairpin. We also noted many P-loop NH resonances that were missing in apo BlaR^S^ became visible in CBAP-acylated BlaR^S^. This suggests CBAP acylation alters the intrinsic microsecond-millisecond exchange sensed by the apo P-loop. This alteration likely reflects P-loop stabilization by hydrophobic contacts between residues F421/W424 and CBAP seen in PDB 3Q7Z [22].

### CBAP-acylation induces slow conformational exchange in the β5/β6-hairpin of BlaR^S^

A unique feature of CBAP-acylated BlaR^S^ compared to other acyl-BlaR^S^ complexes is the direct observation of two states as shown by the protein backbone nuclei. In particular, many residues show a doubling of amide NH cross-peaks, which are connected by exchange cross-peaks in 2D ^1^H^N^-^15^N EXSY spectra; thus, these resonances directly reveal dynamic inter-conversion between distinct states (Fig 3, orange spheres). Unusually large ^1^H^N^ and ^15^N chemical shift differences between the exchange-coupled states occur in the β5-β6 loop (Fig 3B, Table 1), with the most prominent differences at G530, V532, and Y536.

**Table 1:** β5/β6 hairpin chemical shift differences and J_eff_(0) values for the two-states of CBAP-acylated BlaR^S^.

	Two-State Chemical Shift Difference (ppm)					J_eff_(0) (ns/rad)	
Residue	^1^H^N^	^15^N	^13^Cα	^13^Cβ	^13^C'	State 1	State 2
**T527**	0.117	0.205	0.209	0.000	0.471	7.19 (0.13)	^c^—
**G528**	0.147	0.137	0.298	—	0.657	8.2 (0.3)	9.2 (0.4)
**T529**	0.090	0.218	0.388	0.149	—	7.80 (0.08)	^c^—
**G530**	0.924	1.276	0.447	—	—	^c^—	^c^—
**I531**	^a^—	^a^—	^b^0.984	^b^1.639	^b^1.613	^a^—	^a^—
**V532**	0.416	3.453	0.269	0.223	0.014	6.42 (0.09)	7.4 (0.4)
**N533**	0.074	0.206	0.089	0.149	0.043	7.1 (0.5)	8.39 (0.07)
**G534**	0.235	0.342	0.209	—	0.114	5.88 (0.18)	5.6 (0.3)
**K535**	0.073	0.411	0.179	0.000	0.129	6.27 (0.16)	7.04 (0.10)
**Y536**	0.220	0.752	0.298	0.298	0.471	6.7 (0.2)	^c^—

Chemical shifts determined from standard triple resonance experiments at 295 K, pH 7.0, 16.4 T. Values reported are the differences between the two states |δ^CBAP,1^ - δ^CBAP,2^|. J_eff_(0) values measured at T(nom) = 293.8 K, pH 7.0, 18.8 T; uncertainty estimates are indicated in parenthesis.

^a^ I531 ^1^H^N^/^15^N resonance not assigned due to exchange broadening.

^b^ Chemical shift values for I531 determined from the V532 cross sections in the HN(CO)CACB and HNCO triple resonance experiments.

^c^ J_eff_(0) value not determined due to spectral overlap or poor signal intensity.

There was no evidence of slow exchange in apo BlaR^S^, and our exploratory ^15^N CPMG relaxation dispersion measurements did not suggest intermediate exchange. However, this does not preclude intermediate to fast chemical exchange. Indeed, our previous spectral density analysis of the backbone NHs indicated elevated transverse relaxation rates for some β5-β6 residues in apo BlaR^S^ [21]; this suggested the apo-state exchange occurred on a faster time-scale that produced flat ^15^N dispersions over the range of CPMG frequencies used (50 s^-1^ < ν_CPMG_ < 1000 s^-1^).

For the slow exchange cross-peaks (Fig 3), we estimated the exchange rate constants by recording a series of heteronuclear 2D ^15^N exchange spectra (EXSY). Representative exchange rectangles include V532 and Y536 in the β5-β6 hairpin (Fig 3B). We fit the well-resolved exchange rectangles of residues G530, V532, and Y536 to a standard two-state model. The mean forward rate constant *k*_*A*→*B*_ and reverse rate constants *k*_*B*→*A*_ were 2.6 ± 0.3 s^-1^ and 2.6 ± 0.2 s^-1^, respectively. We found the rate constants were similar among the different residues (S2 Table). The simplest explanation for such similarity is that the exchange peaks reflect the same global process.

To investigate the source of chemical exchange in CBAP-acylated BlaR^S^, we examined the backbone heteronuclear chemical shifts. ^13^C^α^ and ^13^C^β^ chemical shifts are acutely sensitive to the backbone φ/ψ torsion angles and, to a much lesser extent, the local environment [44,49,50]. Table 1 reports the two-state chemical shift difference of the various backbone nuclei for residues in the β5-β6 hairpin. Clearly, residues with the largest ^15^N/^1^H chemical shift differences have corresponding differences in their ^13^C^α^ and ^13^C^β^ nuclei. These differences strongly suggest CBAP-acylated BlaR^S^ has at least two distinct β5-β6 hairpin conformations. The spatially proximal Ω-loop residues W475 and M476 also show significant ^13^C chemical shift differences between their exchange states. ^1^H^N^ and ^15^N chemical shifts also depend on backbone torsion angles, especially the psi (Ψ) torsion angle of the preceding residue Ψ_i-1_ [44,50]. As stated, G530, V532, and Y536 have prominently different ^15^N/^1^H chemical shifts between the two states. Combined, these observations indicate the slow exchange process must involve interconversion between distinct β5-β6 hairpin conformations, and highlight an apparent ‘hinge-like’ role of residues V532 and Y536.

### CBAP ligand dynamics is responsible for the β5/β6-hairpin conformational exchange

Amide ^1^H^N^ chemical shifts are markedly sensitive to environmental factors such as ring currents and hydrogen bonding in addition to backbone torsion angles [50,51]. Importantly, residues apart from the β5-β6 and Ω-loop residues described above do not demonstrate state-dependent heteronuclear ^13^C/^15^N chemical shifts (S1 Table). Alternatively, the distinct ^1^H^N^ chemical shifts and slow chemical exchange could reflect the mobility of bound CBAP instead of conformational heterogeneity of the BlaR^S^ backbone. This consideration motivated us to investigate the bound-state ligand flexibility. Importantly, such flexibility is not apparent in the CBAP-acylated BlaR^S^ crystal structure (PDB 3Q7Z), which depicts a single, well-defined conformation of the bound ligand (Fig 1).

We explored the flexibility of bound CBAP using a U-[^2^D]-BlaR^S^ sample in a 99.9% D_2_O buffer background. Comparisons of ^1^H-^1^H ROESY spectra of isolated versus covalently bound CBAP show distinct aromatic and methyl ^1^H chemical shifts, and thus allowed us to investigate the possibility of bound CBAP exchange dynamics.

**Fig 4:**
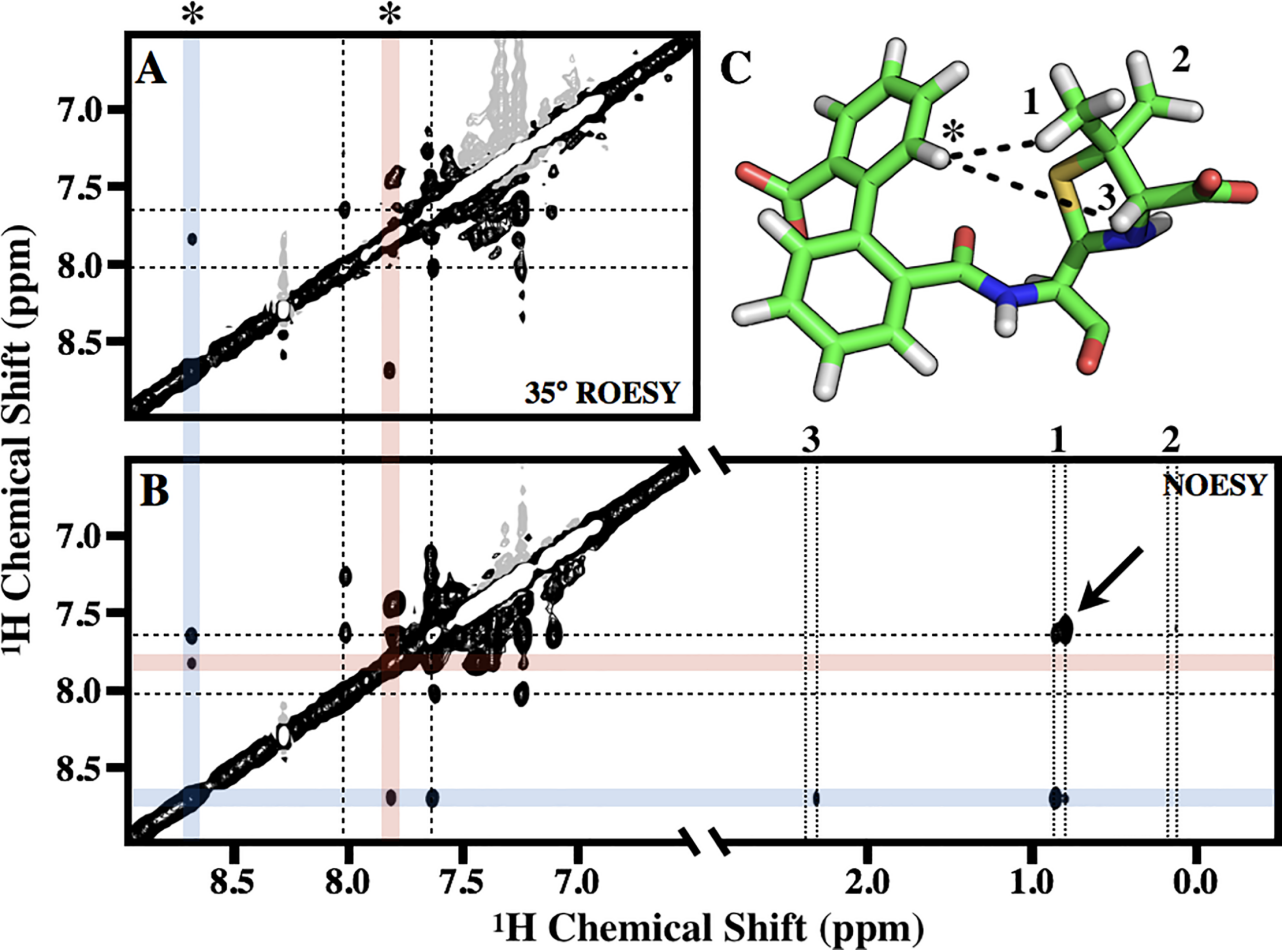
Dynamics of bound CBAP. (A) 35° tilted adiabatic off-resonance ^1^H-^1^H ROESY of bound CBAP using U-[^2^D,^12^C,^14^N] BlaR^S^. Spectra was recorded at 18.8 T and T(nom) = 293.8 K using a 20 ms spin lock (ν = ω_1_/2π = 7,000 Hz). (B) ^1^H-^1^H NOESY spectrum of bound CBAP. (C) Stick diagram of bound CBAP (PDB 3Q7Z); dotted lines indicate proton-proton distances less than 3.5 Å consistent with NOEs. The asterisk is the 6’ proton of the carboxyphenyl. Numbers ‘1’ and ‘2’ are the thiozolidine methyl protons; the ‘3’ indicates the methine proton. The same asterisk and numbers are in the ROESY (A) and NOESY (B) spectra, indicating the corresponding resonances. In both (A) and (B), blue and red shaded lines highlight the two states corresponding to the 6’ proton of the carboxyphenyl (asterisk). The dashed lines indicate the second aromatic proton in slow exchange. In (B), the vertical dotted lines highlight the two states for the thiozolidine methyl (‘1’ and ‘2’) and methine (‘3’) resonances.

Accordingly, we used off-resonance ROESY methods to explore the ^1^H aromatic resonances [45]. We applied a tan/tanh adiabatic spinlock [52] at a 35° tilt angle, which cancels NOE/ROE cross-peaks, and thus distinguishes cross peaks caused by slow chemical exchange. The spectra showed two examples of such exchange: (1) resonances at 8.7 and 7.825 ppm (Fig 4A, red and blue shaded lines); and (2) resonances at 8.0 and 7.625 ppm (Fig 4A, dashed lines). The differences in chemical shift, given the 18.8 T external magnetic field, indicates exchange rates < 300 s^-1^, which is consistent with the rate constants determined by heteronuclear ^15^N-^1^H EXSY above. A ^1^H-^1^H NOESY spectrum of acyl CBAP indicates the ROESY-detected exchange process in Fig 4B corresponds to dynamic inter-conversion between distinct conformations of bound CBAP. For example, the exchange rectangle between 8.7 and 7.825 ppm of Fig 4B corresponds to the 6’ proton of the carboxyphenyl–denoted as an asterisk in Fig 4C–hopping between two distinct conformations. Specifically, the 8.7 ppm resonance (blue lines) gave NOE cross peaks consistent with the crystal structure of bound CBAP [22], and indicates close proximity to (1) the 5’ proton of the carboxyphenyl, (2) one of the thiozolidine methyls, and (3) the thiozolidine methine. These cross peaks are absent in the 7.825 ppm resonance (red lines). This suggests a different set of inter-proton distances that results from a conformational change of the CBAP biphenyl group.

The CBAP methyl ^1^H resonance described above has two peaks; the resonance less than 0.1 ppm upfield corresponds to the second state and demonstrates an alternate NOE pattern: (1) to the thiozolidine methine and (2) to a different aromatic proton slightly upfield of the 7.625 ppm resonance (Fig 4B, arrow). The assignment of this aromatic proton remains ambiguous due to spectral overlap. We speculate this corresponds to a proton of the phenyl directly bonded to the penicillanic acid.

### Acylation by CBAP alters the functional dynamics of BlaR^S^

We previously reported the backbone dynamics of apo BlaR^S^, and the changes caused by acylation with PenG [21]. Here, we discuss the changes caused by CBAP, drawing attention to aspects unique to this ligand. The covalent acylation of BlaR^S^ by CBAP had minimal effects on the overall rotational behavior of the protein. The trimmed means and standard deviations of the ^15^N relaxation rates (T_nom_ = 293.8K and 18.8T) of CBAP-acylated BlaR^S^ are 〈*R*_1,*CBAP*_〉 = 0.49 ± 0.03 s^-1^, 〈*R*_2,*CBAP*_〉 = 30.3 ± 1.1 s^-1^, and 〈*ssNOE*_*CBAP*_〉 = −0.22 ± 0.05. Reduced spectral density mapping resulted in a trimmed mean of 7.4 ± 0.3 ns/rad for $\langle J_{eff}^{CBAP}(0)\rangle$. These values are, within error, the same as those found for apo BlaR^S^ ($\langle J_{eff}^{apo}(0)\rangle =7.6\pm 0.5\;ns/rad$) indicating CBAP does not alter the weak dimerization of the sensor domain *in vitro* [21]. Spectral density values and their respective scatter plots are in S3 Table and S3 Fig, respectively.

**Fig 5:**
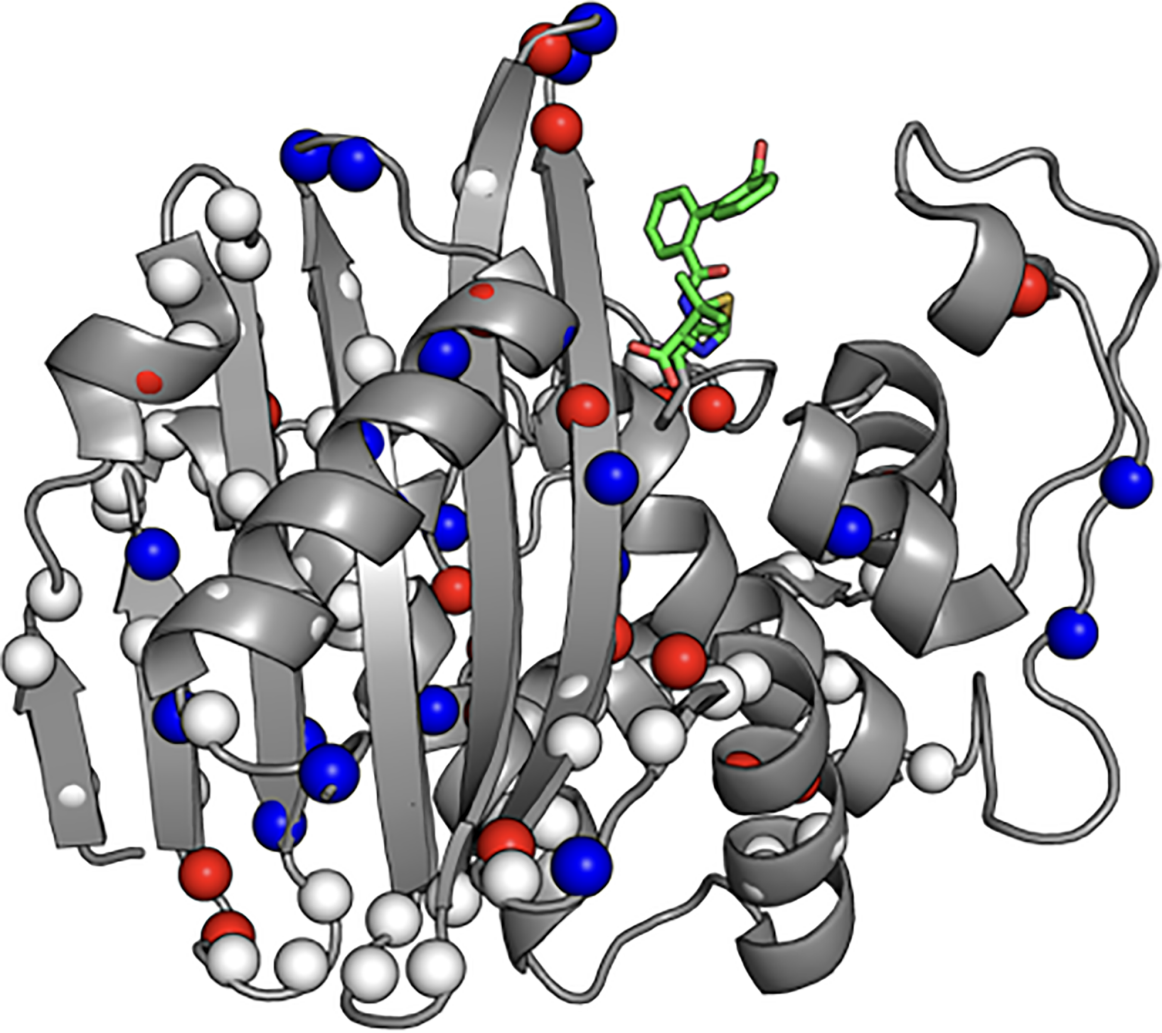
Changes in J_eff_(0) due to acylation of BlaR^S^ by CBAP. Comparison of μs-ms or ps-ns dynamics between apo and CBAP-acylated BlaR^S^ using reduced spectral density mapping. The parameter J_eff_(0) was compared using a dimensionless ΔJ(0) ratio. Spheres indicate residues whose assignments are both known and can be compared between apo and CBAP-acylated BlaR^S^. Residues whose dimensionless ΔJ(0) ratios greater than two standard deviations of the core average are indicated by red (positive) and blue (negative) spheres. Positive ratios correspond to enhanced μs-ms or reduced ps-ns dynamics; negative ratios correspond to reduced μs-ms or enhanced ps-ns dynamics.

Acylation induced site-specific changes to both picosecond-nanosecond and microsecond-millisecond dynamics throughout the protein. These changes were quantified by comparing $J_{eff}^{CBAP}(0)$ to $J_{eff}^{apo}(0)$ using Eq 4. Residues whose dimensionless ratio are, within error, outside one standard deviation (0.03 ns/rad) of the baseline are depicted in Fig 5. Red spheres correspond to those sites whose $J_{eff}^{CBAP}(0)>J_{eff}^{apo}(0)$; this indicates either a decrease in picosecond-nanosecond dynamics, an increase in exchange R_ex_ contributions, or both. Conversely, blue spheres correspond to those sites whose $J_{eff}^{CBAP}(0)<J_{eff}^{apo}(0)$ which indicates either an increase in sub nanosecond bond motions, a decrease in exchange R_ex_ contributions, or both. Several core and binding pocket residues whose $J_{eff}^{apo}(0)$ indicate the presence of exchange contributions demonstrate a decreased $J_{eff}^{CBAP}(0)$; this suggests a decrease in the contribution of R_ex_ to their spectral density value. Other prominent changes in site-specific dynamics include residues in the β5/β6 hairpin, the P-loop, and the hinge regions of the Ω-loop. These areas exhibited both increases and decreases of $J_{eff}^{CBAP}(0)$. Although these changes are more difficult to interpret, these changes have significant biological implications. An alternate view of Fig 5 and the J_eff_(0) outliers for apo and CBAP-acylated BlaR^S^ can be found in S4 Fig.

Resolved exchange peaks in CBAP-acylated BlaR^S^ presented the unique opportunity to directly characterize state-specific dynamics. For example, we compared the state-specific J_eff_(0) on a residue-by-residue basis. Interestingly, β5-β6 hairpin residues showed nonequivalent state-specific J_eff_(0) (Table 1). This indicates these residues have distinct dynamics within the individual conformational states. Most residues outside the β5-β6 hairpin, excluding some Ω/P-loop residues, with resolved exchange peaks showed little to no difference in their J_eff_(0) values (S3 Fig and S3 Table). These differences are much smaller in magnitude compared to the β5-β6 hairpin. These residues make minimal to no contact with the phenyl rings of CBAP, making secondary structural differences between the two states unlikely. Therefore, it is unsurprising that these residues have approximately equal two-state J_eff_(0) values. This corroborates our interpretation that their slow exchange reflects the intrinsic dynamics of the CBAP biphenyl group.

## Discussion

Protein binding typically initiates or propagates the chemical signals sustaining cell survival and adaptation. Mounting experimental evidence indicates that protein binding mechanisms rely on intrinsic conformational dynamics [53–56]. This study serves as a prime example, describing the consequences of the β-lactam CBAP (MW = 442) binding to and acylating a conserved serine within the active site of BlaR^S^ (MW = 29,000). BlaR^S^ is the extracellular β-lactam sensor domain of BlaR1, a transmembrane sensor/transducer protein regulating the β-lactam resistance response in *S*. *aureus* [9,14–15]. β-lactam binding to BlaR^S^ initiates the β-lactam resistance response of methicillin-resistant *S*. *aureus* [25,57]. Here, we show that CBAP alters the conformational dynamics of the BlaR^S^ active site and neighboring segments previously implicated in transmembrane signal transduction [20–21]. These findings strengthen the growing view that investigations of conformational dynamics are important for understanding and predicting the consequences of protein binding [53–56].

NMR spectroscopy has played a prominent role in this context, as it can identify the amino acid cohorts engaged in conformational exchange related to binding and/or catalysis [58–60]. Typically, the exchange is sufficiently rapid such that an exchanging spin system (e.g. a ^1^H-^15^N bond) produces a single broadened resonance. Accordingly, methods promoting direct observation of the NMR resonances of the exchanging are of interest. An exemplary method is the recent study of Wolf-Watz and co-workers that engineered in disulfide bonds to adenylate kinase, to resolve open questions concerning the role of conformational selection in substrate recognition [26].

In this work, we show an alternative approach that bypasses mutation of the protein. We demonstrate how a small-molecule can act as a chemical “time-scale shifter” to expose otherwise obscure conformational dynamics in proteins. In particular, we show how binding of the β-lactam CBAP to BlaR^S^ exposes previously hidden active site dynamics of the latter. Bound CBAP reveals slow conformational exchange between distinct active-site conformations, enabling direct observation of resonances from the interconverting conformations. Of note, being a β-lactam, CBAP is a natural ligand of BlaR^S^.

Previous studies of BlaR^S^ provided convincing evidence that BlaR^S^ has significant internal flexibility, sampling different local conformations at sites relevant for β-lactam acylation. Moreover, β-lactam acylation perturbs BlaR^S^ sampling of those conformations [12,15,20,21]. However, these studies have not resolved these from one another, and so our understanding of their response to β-lactam acylation and their impact on signal transduction has remained limited.

Our NMR studies here take a significant step forward in resolving these conformational states, and strengthen our hypothesis that BlaR^S^ signal transduction involves propagated changes in flexibility [21]. Thanks to slow-exchange, we can directly observe resonances corresponding to distinct conformations of the BlaR^S^ antibiotic binding pocket, and dynamic transitions between them. Below, we discuss these conformations in more detail, and hypothesize on their contribution to signal transduction.

### The β5/β6 hairpin of acylated BlaR^S^ adopts multiple conformations

CBAP-acylation attenuates the conformational sampling of the β5/β6 hairpin. Chemical shift indexing (CSI) predicts the β5/β6 hairpin adopts a two-residue class 2 hairpin. This is consistent with the CBAP-acylated BlaR^S^ crystal structure. Further, the CBAP-acylated BlaR^S^ crystal structure (PDB 3Q7Z) indicates the β5/β6 hairpin contains a β-bulge between G530 and N537/N538 (S5A Fig) [61]. As stated above, residues G530, V532, and Y536 have the largest ^1^H^N^-^15^N EXSY exchange squares (Fig 3B), while the I531 resonance is exchange broadened. Resonances for N537 and N538 are missing. Exchange broadening of N537 and/or N538 due to microsecond/millisecond dynamics is a plausible reason for these missing assignments. However, this is admittedly speculative given that most β6 resonances are not resolved due to the poor hydrogen/deuterium back exchange of core BlaR^S^ residues [21]. Nevertheless, these data highlight the apparent hinge role of these residues in the β5/β6 hairpin.

N533, G534 and K535 have smaller ^15^N and ^13^C chemical shift differences between states (Table 1) compared to hinge residues G530, V532, I531, and Y536. This suggests the two-residue class 2-hairpin structure is maintained in both β5/β6 hairpin conformations. Thus, the conformational states in CBAP-acylated BlaR^S^ likely differ by a “kink” of the secondary structure at the β-bulge. Unfortunately, missing assignments for β5 residue I531 and β6 residues, especially those of N537 and N538, impede the derivation of individual β5/β6 hairpin conformations from chemical shifts. Conceivably, combination of chemical shifts with other local conformational information (e.g. NOEs and solvent accessibility) would permit more detailed structural models of the two conformations. Exploring this possibility is the subject of future work.

### Rotation of the CBAP biphenyl requires reorganization of the β5/β6 hairpin

There is no evidence of bound ligand dynamics in the crystal structure of CBAP-acylated BlaR^S^ (PDB 3Q7Z). However, active-site residues apart from the β5/β6 hairpin and Ω-loop residues W475/M476 display slow exchange peaks solely resolved in the ^1^H^N^ chemical shift dimension. That is, the two resonances for these active-site residues have indistinguishable ^15^N and ^13^C chemical shifts suggesting backbone conformational change is not responsible for the slow exchange at these sites.

In as much, we detected exchange cross-peaks between resonances unique to bound CBAP in a ^1^H-^1^H off-resonance ROESY spectrum (Fig 4A). That is, these aromatic resonances corresponded to the same CBAP biphenyl proton ‘hopping’ between two states. This is not the first evidence of ligand dynamics in the BlaR^S^ antibiotic binding pocket. The two asymmetric units of the penG-acylated BlaR^S^ crystal structure (PDB 1XA7) show two distinct orientations of the penG phenyl substituent [16]. Yet, in our previous penG-acylated BlaR^S^ work, we observed only one ^1^H^N^-^15^N resonance per residue [21]. If the penG ring flip occurs *in vitro*, then it must be in the intermediate-to-fast exchange regime such that the chemical shifts are a population weighted average.

**Fig 6:**
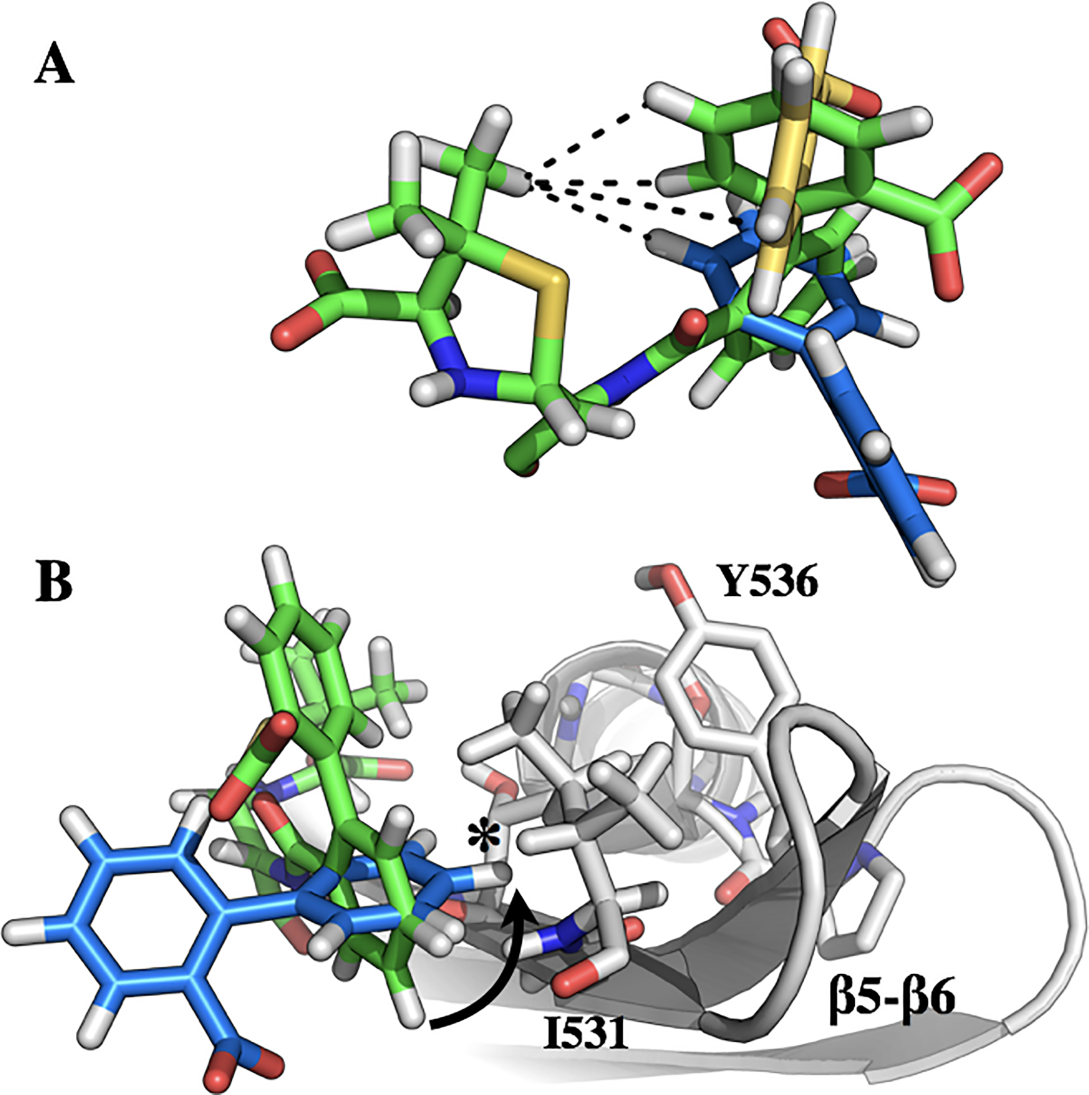
Model of the CBAP ring flip in CBAP-acylated BlaR^S^. (A) The degrees of rotational freedom of the CBAP biphenyl substituent. Green sticks correspond to the bound conformation of CBAP in the x-ray crystal structure, PDB 3Q7Z. Yellow and blue sticks correspond to racemization of the biphenyl and rotation of the entire biphenyl unit, respectively. (B) Proposed rotation of the CBAP biphenyl demonstrates a steric clash–indicated with an asterisk–with the β5/β6 hairpin that would necessitate a conformational change.

The precise mode of ligand dynamics in CBAP-acylated BlaR^S^ is not immediately clear. The low RMSD of the thiazolidine ring in the PenG and CBAP acyl-protein crystal structures shifted our focus to the biphenyl-carboxylic acid R-group, which distinguishes CBAP from other penicillins. This moiety has three degrees of freedom: (1) rotation of the carboxylic acid, (2) racemization of the biphenyl dihedral angle, and (3) rotation of the biphenyl as a single unit. The two CBAP conformations have differing NOESY patterns, which immediately rules out rotation of the carboxylic acid as the source of slow exchange. Fig 6A depicts the second and third rotational degrees of freedom.

2,2’-disubstituted biphenyls have large activation energy barriers that largely prevent racemization of the biphenyl dihedral angle at room temperature; e.g. the barrier in 2,2’-dimethyl biphenyl is approximately 16.7 kcal/mol [62]. The 2,2’-biphenyl substitutions in CBAP (carboxylic acid and penicillanic acid) are larger than methyl groups and likely increases the racemization energy barrier further. This lowers the likelihood racemization is responsible for slow exchange. Therefore, the more probable motion is that of the biphenyl moving as a single unit (Fig 6, blue sticks).

The NOESY spectrum further supports the CBAP biphenyl substituent as being responsible for the exchange process reported here in, and that the motion is likely characterized as a rotation of the biphenyl unit as whole. Racemization of the biphenyl torsion angle would result in the loss of NOE cross-peaks between aromatic protons and methyl protons (interproton distances > 5 Å). Rotation of the entire biphenyl group would also result in this loss, but would yield a new NOE cross peak between the thiazolidine methyl and the phenyl directly attached to the penicillanic acid. Although spectral overlap precludes unambiguous assignment of these phenyl protons, the appearance of this unique NOE supports this interpretation (Fig 4B, arrow).

Rotation of the biphenyl creates a steric clash with I531 of BlaR^S^ (Fig 6B). This corresponds to the hairpin’s hinge region (G530/I531) as discussed above. Therefore, conformational change of the β5/β6 hairpin to alleviate the steric clash introduced by CBAP likely describes the exchange process in CBAP-acylated BlaR^S^.

Interestingly, the deacylation rate of CBAP is significantly reduced compared to penG in both BlaR^S^ and class D β-lactamases, and concomitantly CBAP induces β-lactamase expression to a significantly larger extent [25]. Furthermore, β-lactam specificity in the structurally homologous class D β-lactamases depends on the β5/β6 hairpin conformation [63–65]**.** It is reasonable to posit that, like class D β-lactamases, the conformational sampling of the β5/β6 hairpin may contribute to the broad specificity and function of BlaR^S^. Furthermore, we suspect the efficiency of signal transduction depends on the extent to which covalently bound β-lactam modifies the conformational exchange of the β5/β6 hairpin (e.g. altered rate constants and/or populations of the exchanging conformational states).

### Helix K and P-loop of BlaR^S^

Acylation by CBAP highlights two additional BlaR^S^ structural motifs that merit scrutiny: (1) the P-loop and (2) helix K.

Many P-loop (residues 403–428) amide resonances are exchange broadened in apo BlaR^S^; that is, the P-loop is intrinsically flexible and undergoing conformational exchange on the intermediate timescale [21]. Distinctively, many of these resonances are no longer exchange broadened and are assigned in CBAP-acylated BlaR^S^ (S4B Fig). Of these, only residues D422, W424, N425, and K426 show evidence of conformational exchange either by slow exchange peaks or enhanced J_eff_(0) values. We note residues H416, K417, H418, Y419, and F421 were still exchange broadened suggesting residual flexibility in the P-loop tip. Nevertheless, the P-loop in CBAP-acylated BlaR^S^ exhibits an altered conformational landscape relative to the apo protein.

We observe acylation by CBAP leads to slow conformational exchange and alterations of sub-nanosecond and microsecond/millisecond dynamics in the N-terminus of helix K and its preceding loop (S4C and S5B Figs). We saw similar perturbations in Helix K of penG-acylated BlaR^S^ [21]. These motifs are adjacent to the β5/β6 hairpin whose perturbations are likely an extension of the altered β5/β6 hairpin dynamics induced by covalently bound antibiotic, and may be mediated by hydrogen bonding between T529 and G565 (S5C Fig). We suspect these perturbations could contribute to signal transduction. Previous reports support this hypothesis; for example, deletion of the terminal helix K of BlaR^S^ confers constitutive induction of β-lactamase expression [16]. Also, we previously reported an interaction between BlaR^S^ and the extracellular loop L2 that is mediated by both the β5/β6 hairpin and helix K [20].

## Conclusion

Until recently, the role of the β5/β6 hairpin in BlaR1 signaling remained obscure. A subsequent investigation found acylating BlaR^S^ with the β-lactam penG alters the intrinsic dynamics of the β5/β6 hairpin. The precise details have remained elusive. The β-lactam CBAP now provides key insights for these changes. Our results characterize a previously undetected conformational change in the β5/β6 hairpin secondary structure that accommodates the biphenyl ring flip of CBAP, highlighting this molecule as a useful tool to study the hidden conformational dynamics of BlaR^S^ and possibly other structurally homologous proteins that bind β-lactam antibiotics.

More broadly, CBAP’s revelations suggest the practical advantage of identifying ligands that serve as dynamic time-scale shifters. Such ligands would serve as chemical tools to better expose the conformations sampled by the protein in solution, thus making the specificity of dynamics clearer and more readily exploited by inhibitor design.

## Supporting information

S1 Fig NMR signature of BlaR^S^ acylation. (A) zoom of residue G534 in the ^15^N-^1^H^N^ HSQC demonstrating the spectroscopic signature of BlaR^S^ acylation by CBAP. Blue is apo BlaR^S^, red is CBAP-acylated BlaR^S^, and black is the same CBAP-acylated BlaR^S^ after ~1 month. (B) Characteristic decrease in the K392 N^ζ^ resonance intensity reflecting decarboxylation. Spectra correspond to a slice through the ^1^H dimension of the ^1^H^N^-^15^N^ζ^ resonance peak in an HSQC and are offset to help the viewer.
(TIFF)

S2 Fig Chemical shift perturbations in CBAP-acylated BlaR^S^. (A) Bar graph of chemical shift perturbations resulting from the acylation of BlaR^S^ by CBAP. Orange bars represent the chemical shift perturbation of the second resonance for residues in slow exchange. (B) Two views of BlaR^S^ with CSPs mapped. Spheres indicate residues whose assignments are known in both apo and CBAP-acylated BlaR^S^. Orange spheres correspond to residues in slow exchange; blue spheres correspond to residues not in slow exchange with significant CSPs.
(TIFF)

S3 Fig Reduced spectral density analysis. Scatter plots of the J_eff_(0) for apo and CBAP-acylated BlaR^S^ (top and middle panel), and the dimensionless ratio characterizing their differences (bottom panel).
(TIFF)

S4 Fig Spectral density analysis mapped onto BlaR^S^. Two views of BlaR^S^ with J_eff_(0) and the dimensionless ratio mapped as colored spheres. (A) apo BlaR^S^ and (B) CBAP-acylated BlaR^S^. Residues whose J_eff_(0) is greater than two standard deviations of the core average are indicated by red (positive) and blue (negative) spheres. (C) The dimensionless ratio mapped onto BlaR^S^. Residues whose dimensionless ΔJ(0) ratios are greater than two standard deviations of the core average are indicated by red (positive) and blue (negative) spheres.
(TIFF)

S5 Fig Zoom ins of helix K and the β5-β6 hairpin. (A) The β5/β6 hairpin has a β-bulge between residues G530 and N537/N538, indicated by the dotted box. (B) Zoom in of the CSPs in the β5/β6 hairpin and helix K. (C) Inter-residue interactions between the β5/β6 hairpin and helix K.
(TIFF)

S1 Table Resonance assignments of CBAP-acylated BlaR^S^. (PDF)

S2 Table EXSY exchange rates for select β5/β6 residues. (PDF)

S3 Table Reduced spectral density J_eff_(0) values and dimensionless ratio. (PDF)
