# Supplementary figures and images for: A gratuitous β-Lactamase inducer uncovers hidden active site dynamics of the Staphylococcus aureus BlaR1 sensor domain
Source: PLoS One. 2018 May 17;13(5):e0197241. doi: 10.1371/journal.pone.0197241 (PMC5957439; doi:10.1371/journal.pone.0197241)

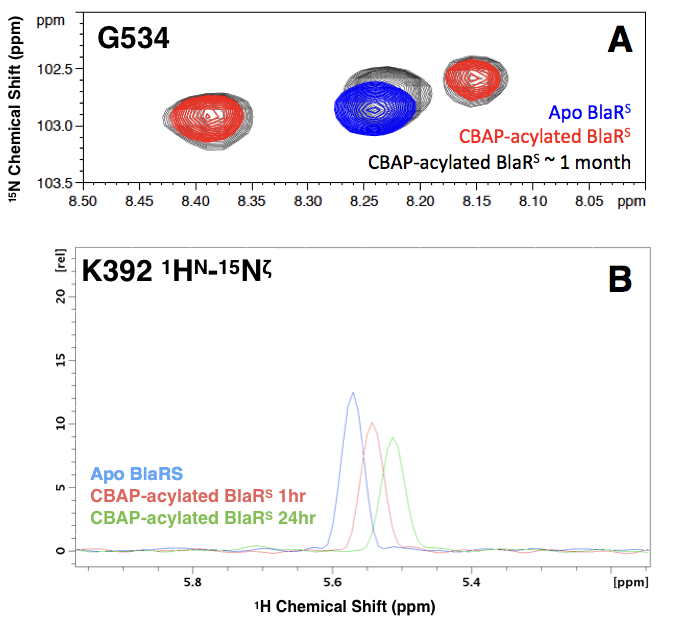

Supplement: S1 Fig — (A) zoom of residue G534 in the 15N-1HN HSQC demonstrating the spectroscopic signature of BlaRS acylation by CBAP. Blue is apo BlaRS, red is CBAP-acylated BlaRS, and black is the same CBAP-acylated BlaRS after ~1 month. (B) Characteristic decrease in the K392 Nζ resonance intensity reflecting decarboxylation. Spectra correspond to a slice through the 1H dimension of the 1HN-15Nζ resonance peak in an HSQC and are offset to help the viewer. (TIFF) [file pone.0197241.s001.tiff]

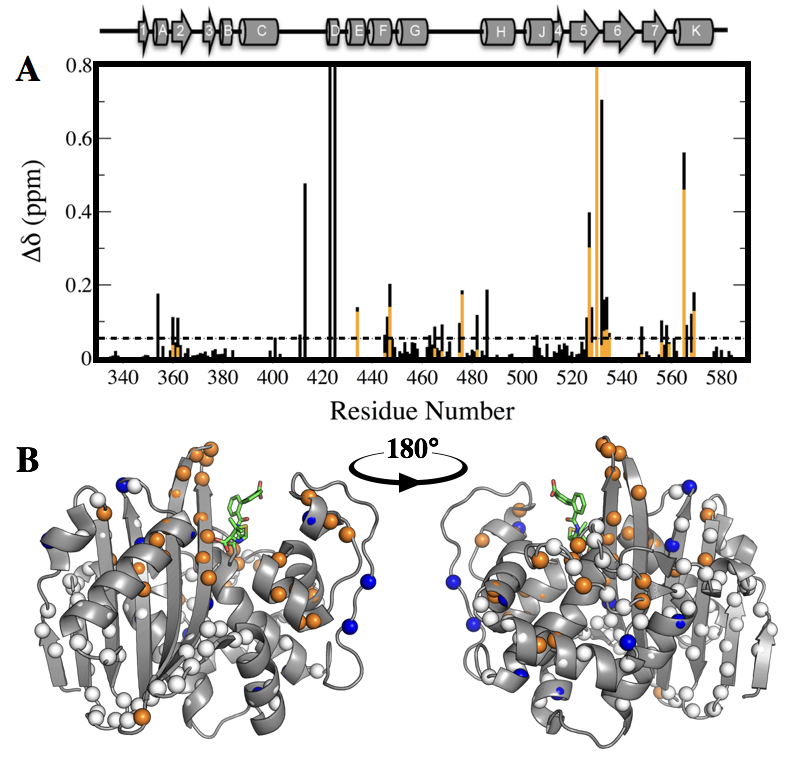

Supplement: S2 Fig — (A) Bar graph of chemical shift perturbations resulting from the acylation of BlaRS by CBAP. Orange bars represent the chemical shift perturbation of the second resonance for residues in slow exchange. (B) Two views of BlaRS with CSPs mapped. Spheres indicate residues whose assignments are known in both apo and CBAP-acylated BlaRS. Orange spheres correspond to residues in slow exchange; blue spheres correspond to residues not in slow exchange with significant CSPs. (TIFF) [file pone.0197241.s002.tiff]

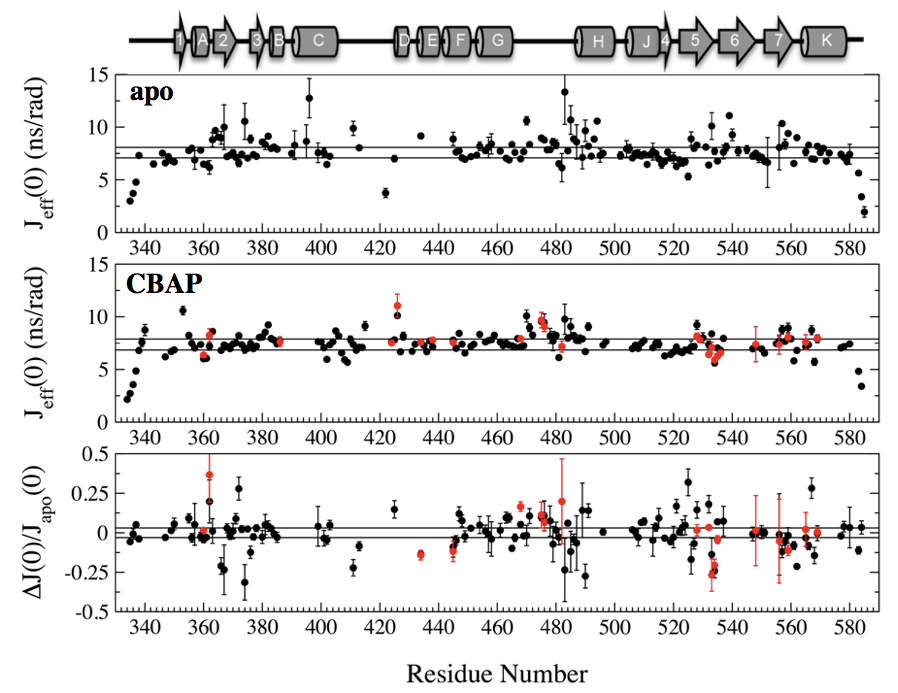

Supplement: S3 Fig — Scatter plots of the Jeff(0) for apo and CBAP-acylated BlaRS (top and middle panel), and the dimensionless ratio characterizing their differences (bottom panel). (TIFF) [file pone.0197241.s003.tiff]

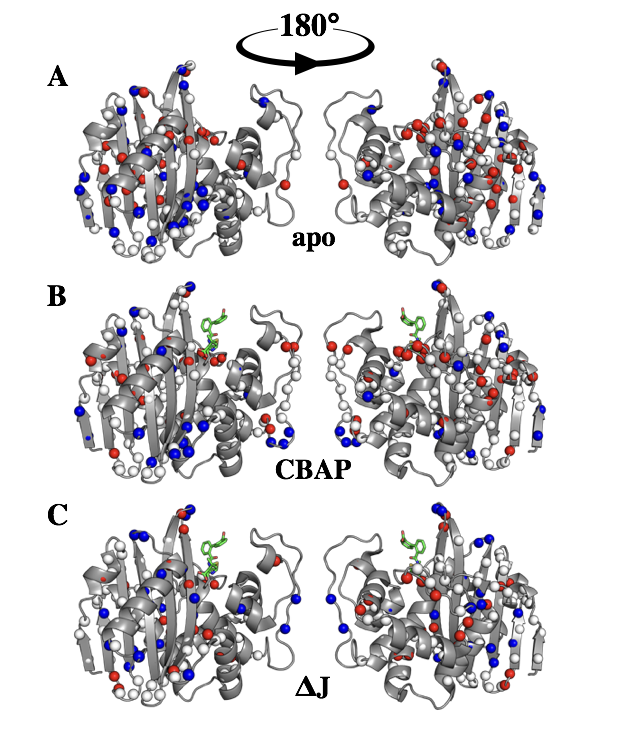

Supplement: S4 Fig — Two views of BlaRS with Jeff(0) and the dimensionless ratio mapped as colored spheres. (A) apo BlaRS and (B) CBAP-acylated BlaRS. Residues whose Jeff(0) is greater than two standard deviations of the core average are indicated by red (positive) and blue (negative) spheres. (C) The dimensionless ratio mapped onto BlaRS. Residues whose dimensionless ΔJ(0) ratios are greater than two standard deviations of the core average are indicated by red (positive) and blue (negative) spheres. (TIFF) [file pone.0197241.s004.tiff]

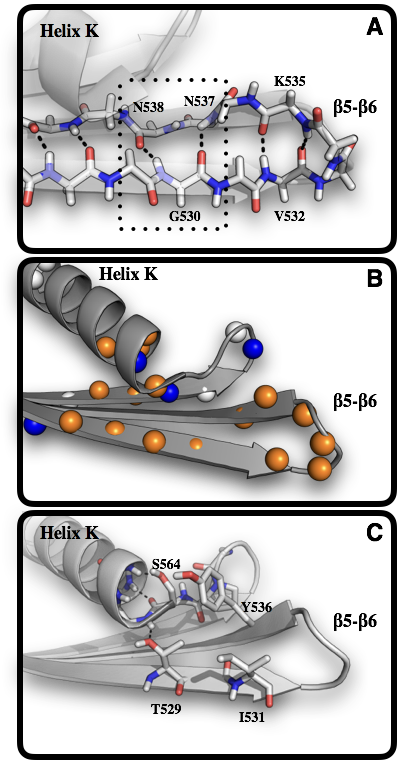

Supplement: S5 Fig — (A) The β5/β6 hairpin has a β-bulge between residues G530 and N537/N538, indicated by the dotted box. (B) Zoom in of the CSPs in the β5/β6 hairpin and helix K. (C) Inter-residue interactions between the β5/β6 hairpin and helix K. (TIFF) [file pone.0197241.s005.tiff]
