# Supplementary material for: A gratuitous β-Lactamase inducer uncovers hidden active site dynamics of the Staphylococcus aureus BlaR1 sensor domain
Source: PLoS One. 2018 May 17;13(5):e0197241. doi: 10.1371/journal.pone.0197241 (PMC5957439; doi:10.1371/journal.pone.0197241)
Supplement: S1 Table — (PDF) [file pone.0197241.s006.pdf]

S1 Table. Resonance assignments of CBAP-acylated BlaR^S

Residue	1HN	15N	13CO	13CA	13CB
--	--	--	174.751	57.724	62.792
334	8.176	123.334	176.706	61.003	37.23
335	7.956	117.319	174.137	61.361	68.903
336	7.839	122.104	176.364	53.55	40.36
337	7.714	120.054	175.308	57.247	37.156
338	7.912	118.344	173.238	51.941	39.987
339	8.934	121.659	176.464	56.383	38.199
347	7.648	118.762	173.566	55.041	39.838
348	8.675	122.172	174.608	53.61	30.821
349	8.734	128.598	176.45	59.661	35.963
350	8.851	127.914	176.135	52.269	45.651
353	9.013	120.053	174.737	57.008	64.133
354	8.631	122.719	178.962	58.886	30.597
355	7.179	119.164	177.363	63.089	36.932
356	7.531	119.574	178.034	61.361	37.677
357	7.736	106.654	175.336	46.516	--
358	8.792	122.445	--	58.26	62.121
359	8.118	119.848	173.68	52.507	39.689
360	7.678	115.61	173.652	56.651	63.835
360_2	7.759	115.582	--	--	--
361	7.883	111.371	170.355	45.621	2.826
362	9.057	111.645	172.738	56.651	66.891
362_2	8.976	111.541	--	--	--
363	8.264	120.805	--	--	43.341
365	9.233	124.77	173.081	53.491	37.752
366	9.819	126.274	174.965	55.816	42.149
367	7.634	120.805	174.665	55.369	63.612
368	8.118	126.479	179.889	56.531	30.001
369	9.951	121.899	179.304	58.529	30.448
370	7.986	115.131	176.007	55.488	31.566
371	7.531	121.078	173.795	53.491	38.05
373	8.382	122.24	--	57.277	40.584
374	9.629	121.01	175.779	55.816	39.019
375	8.895	120.942	175.122	60.585	42.223
376	9.752	128.784	174.009	55.101	39.391
377	8.822	126.274	173.78	51.702	33.951
378	8.837	124.36	179.447	59.363	27.393
379	8.616	119.711	180.104	59.661	30.523
380	7.032	120.053	179.732	58.26	28.213
381	8.602	117.865	--	61.599	--
382	6.856	119.574	175.936	56.591	29.927
383	7.34	123.129	2.826	56.204	31.417
384	8.054	121.528	174.351	--	29.778
385	8.142	120.276	175.322	56.144	42.745

**386**	9.585	117.934	176.621	2.826	63.537
**386_2**	9.639	117.596	--	--	--
**399**	8.675	107.27	175.95	46.963	--
**400**	8.382	124.36	181.588	56.71	40.36
**401**	9.071	123.744	177.42	57.485	41.925
**402**	7.824	111.85	175.408	54.653	28.362
**403**	7.531	113.627	174.865	56.83	25.232
**404**	8.426	120.842	176.592	63.596	37.752
**405**	6.724	109.731	173.909	58.529	40.807
**406**	7.604	117.045	174.066	52.656	38.944
**407**	8.396	117.66	176.849	55.667	39.168
**408**	8.08	119.602	176.692	57.038	28.883
**409**	7.854	116.84	174.451	52.895	38.348
**410**	9.056	122.581	--	56.68	62.047
**411**	8	124.428	177.334	55.995	30.076
**412**	8.954	121.352	175.251	55.22	36.783
**413**	8.587	118.481	173.481	58.171	63.015
**414**	8.631	124.018	175.336	55.16	27.617
**415**	6.871	127.231	--	52.06	36.261
**422**	8.609	126.093	--	57.366	39.689
**422_2**	8.523	125.925	--	--	--
**423**	8.734	118.754	177.991	53.312	16.885
**424**	8.088	116.567	177.277	56.353	28.809
**424_2**	8.05	116.547	--	--	--
**425**	7.274	123.089	173.195	51.494	35.963
**426**	7.003	111.986	174.937	54.952	32.312
**426_2**	6.944	111.929	--	--	--
**427**	8.47	120.258	174.537	56.71	28.958
**428**	7.956	116.703	175.293	52.269	31.268
**429**	7.893	120.531	174.708	52.06	41.329
**430**	7.956	117.865	177.763	58.38	39.019
**430_2**	8.03	117.956	--	--	--
**431**	7.355	114.447	178.605	56.293	36.559
**432**	8.587	114.106	177.791	65.266	67.636
**433**	8.558	125.916	180.532	53.461	16.96
**433_2**	8.592	125.951	--	--	--
**434**	8.212	118.293	179.304	57.157	31.492
**434_2**	8.243	118.259	--	--	--
**435**	8.176	118.344	177.477	58.499	27.617
**436**	7.634	111.44	172.767	52.358	38.05
**437**	7.414	122.377	171.411	58.26	61.376
**438**	8.442	117.897	176.535	60.943	27.766
**438_2**	8.353	118.079	--	--	--
**445**	6.842	117.524	177.177	65.474	36.634
**445_2**	6.77	117.323	--	--	--
**446**	7.795	112.533	177.377	61.211	62.419
**447**	8.03	115.678	177.563	56.054	40.36
**447_2**	7.945	115.486	--	--	--

**448**	7.502	115.473	175.707	55.011	30.001
**449**	7.852	123.306	175.251	58.499	37.603
**451**	8.704	125.863	177.292	59.81	31.045
**452**	8.514	113.901	178.077	55.727	36.41
**453**	6.959	122.172	176.592	60.526	35.889
**454**	7.59	120.19	--	66.249	--
**455**	8.587	122.735	180.732	54.952	16.885
**456**	7.384	116.498	176.335	66.249	67.636
**457**	7.868	120.285	178.89	57.366	26.871
**458**	8.822	116.772	179.59	58.052	38.05
**459**	7.384	118.754	179.318	59.065	30.821
**460**	7.908	120.483	178.548	58.409	--
**461**	8.475	116.634	178.219	54.832	42
**462**	7.472	116.43	174.737	53.103	35.814
**463**	8.088	118.753	173.495	58.558	37.23
**464**	6.504	113.285	173.88	45.413	--
**465**	8.519	119.998	175.707	52.09	36.783
**465_2**	8.603	120.344	--	--	--
**466**	8.22	113.08	173.424	58.052	27.989
**467**	7.003	117.045	175.436	52.388	38.348
**468**	8.455	125.658	176.792	53.819	40.807
**468_2**	8.392	125.383	--	--	--
**469**	7.78	106.313	175.75	46.724	--
**470**	9.101	123.145	174.408	58.081	62.568
**471**	9.392	123.676	174.722	59.125	37.528
**472**	7.835	120.779	2.826	54.385	29.852
**475**	6.446	121.078	172.524	57.724	28.362
**475_2**	6.492	121.54	--	--	--
**476**	6.519	120.736	--	52.284	--
**476_2**	6.506	120.731	--	--	--
**478**	8.153	121.949	176.15	53.491	42.596
**479**	9.002	122.424	175.836	60.943	62.121
**480**	7.135	126.137	176.721	55.369	43.117
**481**	8.176	122.24	173.181	54.385	37.752
**482**	9.731	120.668	171.639	61.152	39.987
**482_2**	9.603	120.649	--	--	--
**483**	7.659	123.833	174.865	57.724	65.475
**484**	9.658	122.924	177.42	57.217	38.87
**485**	8.878	117.798	178.376	58.171	40.509
**486**	7.208	114.584	177.078	58.499	30.15
**487**	8.191	116.361	179.19	59.81	25.828
**488**	6.68	116.567	175.336	66.13	30.896
**489**	7.24	118.998	177.848	65.862	38.87
**490**	8.939	116.567	178.662	65.266	30.597
**491**	6.9	120.531	177.363	57.277	37.603
**495**	7.722	112.283	180.432	55.816	30.97
**496**	8.484	116.156	178.219	56.054	28.511
**497**	7.466	116.599	175.593	55.279	--

**505**	7.907	124.786	179.19	54.713	17.109
**506**	7.898	117.182	178.548	60.168	31.417
**507**	8.264	117.455	177.834	55.488	37.156
**508**	7.883	123.041	179.219	58.588	26.871
**509**	8.294	121.762	180.318	57.366	39.987
**510**	8.616	116.567	175.936	62.821	61.525
**511**	7.88	114.653	176.264	61.897	--
**512**	7.471	117.875	173.952	60.943	63.313
**513**	7.282	120.6	176.107	53.61	43.192
**514**	6.812	121.147	176.664	56.83	39.466
**515**	9.115	129.555	176.535	61.48	38.348
**516**	7.384	115.815	172.553	54.952	34.473
**517**	8.367	121.215	173.823	54.415	34.696
**518**	8.344	122.771	174.109	51.613	38.124
**519**	8.822	117.934	176.764	58.38	27.766
**520**	8.466	117.589	176.535	55.935	32.982
**521**	7.458	115.336	172.239	56.382	39.242
**522**	8.308	115.883	173.895	54.355	33.057
**523**	8.91	125.932	172.553	54.206	44.459
**524**	8.998	127.914	175.579	55.101	39.689
**525**	8.778	106.039	169.741	47.112	--
**526**	8.308	117.182	--	52.269	34.994
**527**	7.956	115.815	177.691	60.794	69.946
**527_2**	7.839	116.02	177.22	60.585	69.946
**528**	8.382	105.697	171.782	47.112	2.826
**528_2**	8.235	105.834	172.439	47.41	2.826
**529**	7.988	118.627	171.582	63.089	72.629
**529_2**	7.898	118.845	--	63.477	72.778
**530**	8.646	113.559	172.11	43.624	--
**530_2**	7.722	112.283	--	43.177	--
**531**	--	--	175.251	60.228	39.317
**531_2**	--	--	173.638	59.244	40.956
**532**	8.426	126.615	176.35	61.092	33.877
**532_2**	8.01	123.162	176.364	61.361	34.1
**533**	9.629	128.735	175.493	53.729	36.41
**533_2**	9.555	128.529	175.45	53.64	36.559
**534**	8.382	102.895	173.181	44.846	--
**534_2**	8.147	102.553	173.067	45.055	--
**535**	7.458	121.283	175.636	53.64	33.504
**535_2**	7.531	121.694	175.507	53.819	33.504
**536**	8.323	124.77	176.892	57.873	35.889
**536_2**	8.543	125.522	176.421	58.171	36.187
**547**	8.778	119.711	173.78	59.244	70.542
**548**	8.822	115.473	175.507	54.713	37.23
**548_2**	8.905	115.362	--	--	--
**550**	7.678	119.369	172.638	52.06	42.074
**551**	9.585	123.402	173.309	55.041	34.845
**555**	8.807	119.164	--	52.299	22.847

**556**	9.534	122.502	--	61.539	70.095
**556_2**	9.466	122.476	--	--	--
**557**	8.588	123.711	172.738	54.504	30.448
**558**	8.206	123.881	174.266	52.567	44.682
**559**	8.646	114.789	173.581	56.263	64.655
**559_2**	8.616	114.843	--	--	--
**560**	7.414	118.822	174.537	53.074	43.043
**561**	8.822	111.371	173.138	45.949	--
**562**	8.338	127.026	173.623	52.954	30.97
**564**	7.413	115.815	174.594	54.952	65.027
**565**	9.189	114.037	175.579	47.738	--
**565_2**	9.275	114.474	--	--	--
**565_3**	9.376	114.421	--	--	--
**566**	8.074	121.42	178.99	57.724	29.778
**567**	7.706	118.287	--	54.385	36.41
**567_2**	7.638	118.501	--	--	--
**568**	8.181	123.031	180.632	54.683	15.991
**568_2**	8.083	122.884	--	--	--
**569**	8.689	127.096	--	59.333	27.393
**569_2**	8.634	127.093	--	--	--
**569_3**	8.554	127.024	--	--	--
**577**	7.927	119.506	180.175	58.678	31.194
**578**	7.707	123.129	178.391	59.125	27.766
**579**	7.875	114.766	176.264	55.935	32.237
**580**	7.384	106.928	174.565	45.055	--
**583**	7.15	117.592	175.308	52.597	38.87
**584**	8.162	108.842	173.167	45.025	--
