# Supplementary material for: A gratuitous β-Lactamase inducer uncovers hidden active site dynamics of the Staphylococcus aureus BlaR1 sensor domain
Source: PLoS One. 2018 May 17;13(5):e0197241. doi: 10.1371/journal.pone.0197241 (PMC5957439; doi:10.1371/journal.pone.0197241)
Supplement: S2 Table — (PDF) [file pone.0197241.s007.pdf]

S2 Table. EXSY exchange rates for select β5/β6 residues

	k_{ab} (s^{-1})	StdDev	k_{ba} (s^{-1})	StdDev
**Y536**	2.29E+00	1.00E-02	2.36E+00	1.30E-02
	2.87E+00	6.00E-02	2.64E+00	4.00E-02
**V532**	2.23E+00	3.00E-02	2.43E+00	5.00E-02
	1.69E+00	7.00E-02	1.97E+00	5.00E-02
**G530**	2.83E+00	5.00E-02	2.77E+00	6.00E-02
