# Supplementary material for: A gratuitous β-Lactamase inducer uncovers hidden active site dynamics of the Staphylococcus aureus BlaR1 sensor domain
Source: PLoS One. 2018 May 17;13(5):e0197241. doi: 10.1371/journal.pone.0197241 (PMC5957439; doi:10.1371/journal.pone.0197241)
Supplement: S3 Table — (PDF) [file pone.0197241.s008.pdf]

S3 Table. Reduced spectral density $J_{eff}(0)$ values and dimensionless ratio.

	CBAP		Apo		CBAP-Apo	
Residue	Jeff(0)	StdDev	Jeff(0)	StdDev	Ratio	StdDev
334	2.16E+0	1.40E-2	--	--	--	--
335	2.72E+0	3.30E-2	2.99E+0	2.91E-3	-5.72E-2	1.11E-2
336	3.56E+0	6.29E-2	3.70E+0	1.09E-1	-8.16E-3	3.30E-2
337	4.87E+0	7.77E-2	4.77E+0	1.11E-2	5.18E-2	1.65E-2
338	6.80E+0	9.49E-2	7.31E+0	1.02E-1	-3.79E-2	1.84E-2
339	7.57E+0	4.01E-1	--	--	--	--
340	8.74E+0	5.27E-1	--	--	--	--
343	--	--	6.50E+0	2.78E-1	--	--
346	--	--	7.52E+0	1.01E-1	--	--
347	6.19E+0	5.15E-2	6.60E+0	1.71E-2	-2.97E-2	8.17E-3
348	--	--	7.21E+0	3.02E-2	--	--
349	6.70E+0	1.01E-1	6.82E+0	4.76E-2	1.40E-2	1.64E-2
350	6.86E+0	9.03E-2	6.70E+0	2.23E-1	5.57E-2	3.67E-2
353	1.06E+1	4.11E-1	--	--	--	--
355	8.24E+0	1.58E-1	7.78E+0	1.44E-1	9.13E-2	2.82E-2
356	7.48E+0	2.64E-2	7.99E+0	2.31E-1	-3.20E-2	2.73E-2
357	7.03E+0	9.83E-2	6.89E+0	8.91E-1	5.28E-2	1.33E-1
359	7.38E+0	5.97E-2	7.81E+0	2.08E-1	-2.36E-2	2.63E-2
360	6.00E+0	1.45E-1	6.48E+0	2.53E-2	-4.15E-2	2.27E-2
360_2	6.37E+0	7.11E-2	--	--	-1.65E-2	1.16E-2
361	6.05E+0	5.80E-2	6.44E+0	4.46E-2	-2.91E-2	1.11E-2
362	7.20E+0	3.82E-1	6.18E+0	6.43E-1	1.98E-1	1.36E-1
362_2	8.25E+0	6.30E-1	--	--	3.35E-1	1.72E-1
363	8.61E+0	1.84E-1	8.80E+0	6.75E-1	1.07E-2	7.80E-2
364	--	--	9.69E+0	6.67E-2	--	--
365	--	--	9.06E+0	7.13E-2	--	--
366	6.81E+0	4.88E-2	8.98E+0	6.15E-1	-2.10E-1	5.22E-2
367	7.33E+0	1.61E-1	1.00E+1	2.13E+0	-2.34E-1	1.57E-1
368	7.17E+0	1.28E-1	7.20E+0	1.59E-1	2.83E-2	2.83E-2
369	6.97E+0	1.71E-1	7.34E+0	1.27E-2	-1.85E-2	2.33E-2
370	7.41E+0	2.35E-1	7.57E+0	2.92E-1	1.07E-2	4.88E-2
371	7.53E+0	1.16E-1	7.13E+0	1.88E-1	8.85E-2	3.23E-2
372	8.21E+0	2.30E-1	6.59E+0	3.34E-1	2.79E-1	7.22E-2
373	7.37E+0	1.57E-1	7.43E+0	1.83E-2	2.32E-2	2.12E-2
374	6.90E+0	3.66E-1	1.06E+1	1.71E+0	-3.14E-1	1.12E-1
375	7.00E+0	6.17E-2	7.06E+0	1.07E-1	2.36E-2	1.74E-2
376	7.48E+0	1.11E-1	8.86E+0	3.85E-1	-1.24E-1	3.87E-2
377	6.97E+0	6.77E-2	7.40E+0	1.33E-1	-2.55E-2	1.92E-2
378	7.21E+0	6.83E-2	7.23E+0	1.37E-1	2.93E-2	2.12E-2
379	8.04E+0	1.65E-2	--	--	--	--
380	8.08E+0	2.87E-1	8.59E+0	2.25E-1	-2.66E-2	4.15E-2
381	8.53E+0	1.37E-1	8.37E+0	5.06E-1	5.19E-2	6.38E-2
382	9.24E+0	2.52E-1	9.15E+0	1.71E-1	4.20E-2	3.34E-2

383	7.94E+0	1.04E-1	7.98E+0	4.19E-2	2.82E-2	1.41E-2
384	7.78E+0	4.33E-2	8.10E+0	3.85E-2	-6.95E-3	7.03E-3
385	7.42E+0	2.55E-1	7.90E+0	9.81E-3	-2.88E-2	3.22E-2
386	7.71E+0	3.83E-1	--	--	--	--
386_2	7.61E+0	4.35E-1	--	--	--	--
390	--	--	7.49E+0	2.59E-1	--	--
391	--	--	8.29E+0	1.37E+0	--	--
395	--	--	8.65E+0	1.58E+0	--	--
396	--	--	1.27E+1	1.87E+0	--	--
399	7.64E+0	1.78E-1	7.57E+0	9.31E-1	4.12E-2	1.26E-1
400	7.61E+0	2.53E-1	--	--	--	--
401	7.07E+0	9.65E-2	7.57E+0	3.91E-1	-3.44E-2	4.99E-2
402	5.95E+0	1.20E-1	6.46E+0	1.42E-1	-4.59E-2	2.75E-2
403	7.34E+0	1.05E-1	7.23E+0	1.84E-1	4.85E-2	2.97E-2
404	7.62E+0	8.77E-2	--	--	--	--
405	8.67E+0	9.48E-2	--	--	--	--
406	8.18E+0	3.69E-2	--	--	--	--
407	6.57E+0	4.62E-2	--	--	--	--
408	5.91E+0	9.52E-2	--	--	--	--
409	5.67E+0	1.94E-2	--	--	--	--
410	7.84E+0	3.36E-1	--	--	--	--
411	7.37E+0	1.05E-1	9.88E+0	6.77E-1	-2.22E-1	5.22E-2
412	6.82E+0	1.03E-1	--	--	--	--
413	7.09E+0	2.02E-1	8.03E+0	1.17E-1	-8.49E-2	2.83E-2
414	7.08E+0	9.00E-2	--	--	--	--
415	9.13E+0	4.28E-1	--	--	--	--
422	--	--	3.75E+0	4.36E-1	--	--
424	7.65E+0	1.96E-1	--	--	--	--
424_2	7.51E+0	1.06E-1	--	--	--	--
425	7.81E+0	1.88E-1	7.00E+0	3.04E-1	1.48E-1	5.54E-2
426	1.01E+1	1.11E-1	--	--	--	--
426_2	1.11E+1	1.09E+0	--	--	--	--
427	6.67E+0	7.71E-2	--	--	--	--
428	8.17E+0	3.65E-1	--	--	--	--
431	6.72E+0	1.45E-1	--	--	--	--
432	7.40E+0	3.37E-2	--	--	--	--
434	7.61E+0	1.30E-1	9.16E+0	1.41E-1	-1.38E-1	1.90E-2
434_2	7.57E+0	2.52E-1	--	--	-1.74E-1	3.03E-2
435	7.27E+0	1.63E-1	--	--	--	--
436	6.68E+0	1.90E-2	--	--	--	--
437	7.71E+0	1.28E-1	--	--	--	--
438	7.13E+0	1.50E-1	--	--	--	--
438_2	7.79E+0	2.01E-1	--	--	--	--
445	7.79E+0	1.11E-1	8.88E+0	6.35E-1	-9.06E-2	6.40E-2
445_2	7.55E+0	2.21E-1	--	--	-1.49E-1	6.57E-2
446	7.01E+0	1.24E-1	7.63E+0	2.16E-1	-4.92E-2	3.07E-2
447	8.43E+0	2.09E-1	7.75E+0	2.53E-1	1.20E-1	4.46E-2
448	7.40E+0	7.68E-2	7.09E+0	2.42E-1	7.60E-2	3.72E-2

449	6.56E+0	2.35E-1	6.94E+0	4.14E-2	-2.24E-2	3.43E-2
451	7.18E+0	7.87E-2	7.20E+0	4.11E-2	2.89E-2	1.23E-2
452	7.40E+0	1.57E-2	--	--	--	--
453	--	--	7.33E+0	1.03E-2	--	--
454	8.33E+0	1.40E-1	8.19E+0	4.13E-1	4.89E-2	5.41E-2
456	7.60E+0	1.24E-1	7.78E+0	2.81E-1	9.57E-3	3.87E-2
457	7.74E+0	6.18E-2	8.08E+0	9.71E-1	-9.53E-3	1.16E-1
458	7.81E+0	2.21E-1	8.41E+0	9.57E-1	-3.93E-2	1.09E-1
459	8.25E+0	1.35E-1	--	--	--	--
461	7.60E+0	2.66E-1	7.72E+0	2.16E-1	1.55E-2	4.40E-2
462	7.26E+0	1.98E-1	--	--	--	--
463	7.50E+0	2.43E-1	7.05E+0	1.72E-2	9.55E-2	3.45E-2
464	7.32E+0	1.47E-1	6.89E+0	1.04E-1	9.42E-2	2.67E-2
465	7.26E+0	7.73E-2	8.36E+0	1.48E-1	-9.89E-2	1.79E-2
466	7.12E+0	1.64E-1	7.60E+0	6.79E-2	-3.13E-2	2.32E-2
467	7.94E+0	1.22E-1	--	--	--	--
468	7.13E+0	1.03E-1	6.99E+0	6.95E-2	5.23E-2	1.79E-2
468_2	7.92E+0	1.99E-1	--	--	1.33E-1	3.06E-2
469	7.25E+0	1.09E-1	7.65E+0	1.74E-2	-2.01E-2	1.44E-2
470	1.01E+1	5.77E-1	1.06E+1	3.94E-1	-1.70E-2	6.49E-2
471	8.97E+0	3.74E-1	8.35E+0	1.27E-1	1.06E-1	4.77E-2
472	8.27E+0	2.11E-1	--	--	--	--
475	9.56E+0	1.94E-1	8.97E+0	4.44E-2	9.88E-2	2.22E-2
475_2	9.69E+0	7.31E-1	--	--	8.05E-2	8.17E-2
476	9.44E+0	8.30E-1	8.77E+0	1.92E-2	1.07E-1	9.46E-2
476_2	9.07E+0	4.79E-1	--	--	3.34E-1	5.46E-2
477	--	--	7.84E+0	1.73E-1	--	--
478	8.19E+0	7.19E-1	7.86E+0	2.12E-1	7.43E-2	9.56E-2
479	7.70E+0	9.19E-1	8.59E+0	3.30E-1	-7.20E-2	1.12E-1
480	8.24E+0	1.20E-1	8.37E+0	4.22E-1	1.58E-2	5.16E-2
481	6.13E+0	2.04E-1	6.52E+0	2.65E-1	-2.82E-2	4.93E-2
482	--	--	6.15E+0	1.34E+0	--	--
482_2	7.17E+0	5.35E-1	--	--	--	--
483	9.77E+0	1.44E+0	1.33E+1	3.07E+0	-2.35E-1	2.00E-1
484	7.98E+0	1.16E-1	7.75E+0	7.42E-2	6.07E-2	1.79E-2
485	9.06E+0	7.53E-1	1.07E+1	1.36E+0	-1.19E-1	1.29E-1
486	8.23E+0	2.53E-1	8.89E+0	1.80E-1	-4.21E-2	3.41E-2
487	7.75E+0	1.65E-1	8.59E+0	1.63E+0	-6.53E-2	1.72E-1
488	7.86E+0	4.12E-1	--	--	--	--
489	7.91E+0	2.17E-1	7.12E+0	1.09E+0	1.42E-1	1.72E-1
490	6.69E+0	2.02E-1	9.66E+0	1.01E+0	-2.75E-1	7.57E-2
491	9.06E+0	3.54E-1	8.18E+0	4.08E-2	1.40E-1	4.36E-2
492	--	--	7.24E+0	2.21E-2	--	--
493	--	--	8.88E+0	2.05E-1	--	--
494	--	--	1.06E+1	1.07E-1	--	--
495	--	--	7.31E+0	6.25E-1	--	--
496	7.32E+0	1.32E-1	7.52E+0	7.95E-2	5.07E-3	2.04E-2
497	7.64E+0	3.09E-2	--	--	--	--

502	--	--	7.31E+0	3.51E-1	--	--
504	--	--	7.94E+0	7.82E-1	--	--
505	--	--	7.94E+0	4.09E-1	--	--
506	6.97E+0	2.04E-2	7.06E+0	1.01E-1	1.88E-2	1.44E-2
507	7.31E+0	1.11E-1	7.51E+0	1.56E-1	5.18E-3	2.51E-2
508	7.00E+0	1.62E-1	7.45E+0	3.98E-1	-2.91E-2	5.46E-2
509	7.49E+0	5.74E-2	7.25E+0	3.55E-2	6.49E-2	9.39E-3
510	7.74E+0	9.27E-2	7.45E+0	1.35E-1	7.16E-2	2.26E-2
511	--	--	6.45E+0	5.20E-2	--	--
512	--	--	7.37E+0	2.38E-1	--	--
513	7.07E+0	1.99E-1	7.68E+0	8.08E-1	-4.80E-2	1.00E-1
514	7.51E+0	1.08E-1	7.48E+0	1.62E-1	3.56E-2	2.60E-2
515	7.41E+0	3.68E-1	6.99E+0	2.19E-1	9.28E-2	6.22E-2
516	--	--	6.49E+0	3.92E-1	--	--
517	6.28E+0	5.53E-2	6.72E+0	1.01E-1	-3.37E-2	1.63E-2
518	--	--	7.63E+0	2.68E-1	--	--
519	6.42E+0	9.55E-2	7.06E+0	2.14E-2	-5.79E-2	1.38E-2
520	6.66E+0	7.77E-2	7.08E+0	5.06E-1	-2.74E-2	6.81E-2
521	7.12E+0	1.16E-1	6.27E+0	2.15E-1	1.68E-1	4.32E-2
522	6.71E+0	2.64E-2	6.90E+0	1.23E-1	4.24E-3	1.78E-2
523	6.60E+0	1.07E-1	6.60E+0	3.07E-1	3.30E-2	4.93E-2
524	6.84E+0	2.13E-1	6.74E+0	3.39E-1	4.71E-2	6.00E-2
525	6.84E+0	1.76E-1	5.31E+0	3.23E-1	3.19E-1	8.49E-2
526	7.11E+0	6.28E-1	8.89E+0	6.65E-1	-1.69E-1	9.25E-2
527	7.19E+0	1.27E-1	7.99E+0	2.71E-1	-6.86E-2	3.44E-2
528	9.23E+0	4.33E-1	8.29E+0	4.85E-2	1.45E-1	5.26E-2
528_2	8.16E+0	2.91E-1	--	--	-1.58E-2	3.55E-2
529	7.89E+0	2.29E-1	--	--	--	--
529_2	7.80E+0	7.89E-2	--	--	--	--
530	8.00E+0	4.85E-1	--	--	--	--
531	--	--	8.10E+0	1.84E-1	--	--
532	7.36E+0	3.54E-1	6.41E+0	1.37E-2	1.80E-1	5.53E-2
532_2	6.42E+0	9.31E-2	--	--	1.72E-3	1.47E-2
533	8.39E+0	6.80E-2	1.01E+1	1.26E+0	-1.38E-1	1.04E-1
533_2	7.07E+0	5.38E-1	--	--	-3.00E-1	1.03E-1
534	5.61E+0	2.70E-1	7.71E+0	3.11E-1	-2.41E-1	4.57E-2
534_2	5.88E+0	1.77E-1	--	--	-2.37E-1	3.84E-2
535	7.04E+0	9.63E-2	6.78E+0	6.33E-2	6.92E-2	1.72E-2
535_2	6.27E+0	1.61E-1	--	--	-7.59E-2	2.53E-2
536	6.82E+0	1.04E-1	--	--	--	--
536_2	6.57E+0	2.31E-1	--	--	--	--
537	7.95E+0	2.38E-1	7.64E+0	6.55E-1	7.35E-2	9.46E-2
538	--	--	8.12E+0	3.68E-1	--	--
539	--	--	1.11E+1	1.99E-1	--	--
540	--	--	9.27E+0	5.52E-1	--	--
542	--	--	7.71E+0	4.28E-1	--	--
545	--	--	7.87E+0	3.72E-1	--	--
547	6.96E+0	1.19E-1	7.25E+0	2.98E-1	-7.68E-3	4.28E-2

548	7.21E+0	9.95E-2	7.53E+0	9.86E-2	-1.06E-2	1.82E-2
548_2	7.39E+0	1.67E-1	--	--	-1.86E-2	2.22E-1
549	--	--	7.08E+0	4.45E-1	--	--
550	6.95E+0	1.37E-1	7.14E+0	2.63E-1	4.35E-3	4.07E-2
551	6.55E+0	5.05E-2	6.76E+0	1.41E-1	2.80E-4	2.16E-2
552	--	--	6.64E+0	2.37E+0	--	--
555	7.45E+0	1.61E-1	--	--	--	--
556	7.71E+0	4.74E-1	8.05E+0	2.11E+0	-1.03E-2	2.57E-1
556_2	7.37E+0	9.24E-1	--	--	-8.49E-2	2.65E-1
557	8.78E+0	3.98E-1	1.03E+1	7.70E-2	-1.19E-1	3.90E-2
558	7.66E+0	1.41E-1	8.39E+0	4.71E-1	-5.49E-2	5.39E-2
559	8.94E+0	4.56E-1	9.40E+0	1.06E-1	-1.74E-2	4.97E-2
559_2	8.04E+0	2.81E-1	--	--	-1.45E-1	3.14E-2
560	7.91E+0	6.68E-2	--	--	--	--
561	5.81E+0	7.79E-2	6.55E+0	1.61E-1	-8.08E-2	2.49E-2
562	6.80E+0	1.32E-1	9.01E+0	1.23E-2	-2.13E-1	1.47E-2
565	7.16E+0	2.02E-1	7.66E+0	3.80E-1	-3.29E-2	5.34E-2
565_2	7.57E+0	7.31E-1	--	--	-1.14E-2	1.07E-1
566	7.34E+0	1.91E-1	8.30E+0	2.02E-2	-8.34E-2	2.31E-2
567	8.73E+0	4.27E-1	6.99E+0	1.08E-1	2.82E-1	6.40E-2
568	5.72E+0	3.34E-1	6.95E+0	1.52E-1	-1.44E-1	5.14E-2
569	7.90E+0	3.86E-1	8.19E+0	2.18E-2	-2.62E-3	4.72E-2
569_2	7.95E+0	2.44E-1	--	--	-2.85E-2	3.00E-2
570	--	--	7.59E+0	2.03E-1	--	--
571	--	--	7.98E+0	5.65E-2	--	--
572	--	--	6.78E+0	7.35E-2	--	--
573	--	--	7.55E+0	1.94E-2	--	--
577	7.05E+0	4.40E-2	7.43E+0	5.04E-3	-1.96E-2	5.95E-3
578	7.16E+0	8.65E-2	7.12E+0	2.19E-1	3.88E-2	3.33E-2
579	--	--	6.71E+0	1.99E-1	--	--
580	7.43E+0	1.70E-1	7.42E+0	9.43E-1	3.33E-2	1.29E-1
583	4.84E+0	8.39E-2	5.65E+0	1.19E-1	-1.12E-1	2.34E-2
584	3.40E+0	1.35E-1	3.39E+0	4.42E-2	3.38E-2	4.19E-2
585	--	--	1.95E+0	5.03E-1	--	--
